# Targeted Gene Delivery into Various Plastids Mediated by Clustered Cell‐Penetrating and Chloroplast‐Targeting Peptides

**DOI:** 10.1002/advs.201902064

**Published:** 2019-10-23

**Authors:** Chonprakun Thagun, Jo‐Ann Chuah, Keiji Numata

**Affiliations:** ^1^ Biomacromolecules Research Team RIKEN Center for Sustainable Resource Science 2‐1 Hirosawa, Wako‐shi Saitama 351‐0198 Japan

**Keywords:** cell‐penetrating peptides, gene delivery, plant science, plastid transformation, signal peptides

## Abstract

The plastid is an organelle that functions as a cell factory to supply food and oxygen to the plant cell and is therefore a potential target for genetic engineering to acquire plants with novel photosynthetic traits or the ability to produce valuable biomolecules. Conventional plastid genome engineering technologies are laborious for the preparation of plant material, require expensive experimental instruments, and are time consuming for obtaining a transplastomic plant line that produces significant levels of the biomolecule of interest. Herein, a transient plastid transformation technique is presented using a peptide‐based gene carrier. By formulating peptide/plasmid DNA complexes that combine the functions of both a cell‐penetrating peptide and a chloroplast‐targeting peptide, DNA molecules are translocated across the plant cell membrane and delivered to the plastid efficiently via vesicle formation and intracellular vesicle trafficking. A simple infiltration method enables the introduction of a complex solution into intact plants, and plastid‐localized transgene expression is expeditiously observed in various types of plastids in differentiated cell types of several plants. The gene delivery technology thus provides a useful tool to rapidly engineer plastids in crop species.

## Graphical abstract

By combining the functions of cationic chloroplast‐targeting and cell‐penetrating peptides, DNA molecules can be efficiently and specifically delivered to various types of plastids—photosynthetic, food storage and supply organelles, in intact plant tissues. This peptide carrier‐based gene transfer technique allows comprehensive functional analysis of corresponding biomolecules and metabolic engineering in plastids.

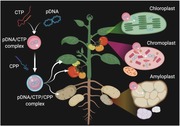

## 1 Introduction

Plastids are organelles found in plant cells and eukaryotic algae and play pivotal roles in photosynthesis and other metabolic processes.^1^ Originally proplastids, these organelles can differentiate into green or nongreen plastids depending on the developmental stage and plant cell type.^1^, ^2^ Each type of plastid has its own characteristics, biological function, and individualized metabolic process in its original cell.^2^ Chloroplasts, the most common plastids found in greenish plant tissues such as developed shoots, leaves, and stems, are responsible for photosynthesis as well as the production of food and oxygen for cells.^1^, ^2^ Another pigmented plastid, the chromoplast, imparts a red‐to‐yellow color to the differentiated cells in fruits and petals of flowers through the synthesis of carotenoids.^3^ The amyloplast is a noncolored plastid found in roots, underground stems, and the storage parts of plants such as tubers and is in charge of the storage of starch.^1^ These differentiated plastids can redifferentiate into other types of plastids based on the cellular mechanism, metabolic function, and developmental stage of the cell in which they exist.^2^, ^3^, ^4^

Apart from its ability to synthesize and accumulate products such as recombinant proteins, small molecules, and bioactive compounds, the plastid also protects biomolecules from the cellular degradation process.^5^ This organelle has become the focus of transplastomic engineering to improve economically important traits or to produce commercially valuable products in the target crop species.^5^, ^6^ The plastome is a 100−200 kbp DNA molecule in the plastid, which harbors 100−120 highly conserved genes and has its own transcriptional and translational regulation system while sharing some plastid‐targeting nuclear‐encoded transcription factors with the nucleus.[qv: 3a] Successful modification of the plastome has been achieved by particle bombardment whereby DNA molecules are transferred across the plant cell boundary and plastid membrane using pressure.[qv: 5a,6a,7] Plasmid DNA (pDNA) can also be directly delivered into the plastid via lipid membrane integration and translocation using polyethylene glycol (PEG) mediated protoplast transformation.^8^ Alternatively, plastid transformation may be accomplished by microinjecting a solution containing exogenous DNA molecules through a sub‐micrometer diameter glass syringe directly into the plastids.^9^ Transplastomic plants can then be established after multiple selection cycles and the subsequent regeneration of transformed plant cells or tissues.^5^ These methods have several major drawbacks, however, as the preparation of plant material is tedious and its regeneration following transformation is time consuming and requires high‐cost experimental instruments; more importantly, these methods are only applicable for limited types and stages of plant material.^5^

**Figure 1:**
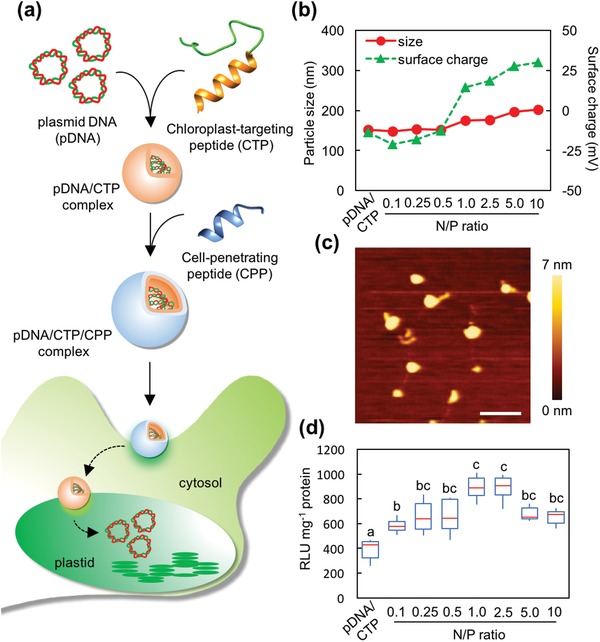
Formulation of the clustered pDNA/CTP/CPP complexes for plastid transformation. a) Transformation of plastids mediated by the clustered CTP/CPP complexes. b) Particle size and surface charge distribution of the pDNA(p*PpsbA::Rluc*)/CTP (KH_9_‐OEP34)/CPP (BP100) complexes at different N/P ratios ranging from 0.1 to 10 and the p*PpsbA::Rluc*/KH_9_‐OEP34 complexes at an N/P ratio of 1.0 (pDNA/CTP). Error bars represent the standard deviation (SD) of the average value from three replicates. c) Morphologies of the clustered p*PpsbA::Rluc*/KH_9_‐OEP34/BP100 complexes at an N/P ratio of 1.0 under AFM imaging. Scale bar = 500 nm. The color bar from 0 to 7 nm represents the height of the complexes. d) *Renilla* luciferase activity in *A. thaliana* leaves syringe‐infiltrated with the clustered complexes formed at different N/P ratios and the complex of p*PpsbA::Rluc*/KH_9_‐OEP34 (pDNA/CTP) at 24 h post‐infiltration. The data are presented in the form of a box plot. Red lines represent the median value (*n* = 6). Maximum and minimum values are presented by the upper and lower bars. Different letters indicate significant differences analyzed by one‐way ANOVA and Tukey's HSD test at *p* = 0.05.

Peptides, short sequences of 30−35 amino acids, have demonstrated their notorious potential to translocate various types of molecules, including nucleic acids, proteins, and small chemicals, across the biological membrane of living cells.^10^ The use of peptides for biomacromolecule delivery in plant cells has progressed significantly in recent years.^11^ Exogenous DNA molecules have been delivered to specific plant intracellular compartments (e.g., mitochondria and chloroplasts) using organelle‐targeting peptides.^12^ Combining the functions of a chloroplast‐targeting peptide (CTP; KH_9_‐OEP34[qv: 12b]) and cell‐penetrating peptide (CPP), we successfully developed a peptide‐based transformation platform to deliver DNA molecules across cell boundaries and subsequently mediate transgene expression in plastids (**Figure** **1**a). Our strategy is simple yet effective; the introduction of a sub‐micrometer‐sized, self‐assembled CPP/CTP complex containing DNA molecules into target plant organs can be achieved by syringe infiltration, injection, or vacuum infiltration, and reporter gene expression in various plastids can be detected after a short incubation period post‐transformation. This new transformation technology can advance plastid modification for plant metabolic engineering and the improvement of photosynthetic traits in a wide variety of crop species.

## 2 Results

### 2.1 Formulation of a Plasmid DNA/CTP/CPP Complex for Chloroplast Transformation

Negatively charged pDNA molecules can electrostatically form a complex with the positively charged CTP KH_9_‐OEP34 (CTP) (Table S1, Supporting Information).[qv: 12b] We first verified the formation of complexes between pDNA encoding *Renilla* luciferase (Rluc) as a reporter (p*PpsbA::Rluc*) (Figure S1a and Table S2, Supporting Information) and the CTP in water at various N/P ratios (defined as the ratio of the moles of amine groups from the peptide to that of phosphate groups from the pDNA) ranging from 0.1 to 25. The particle sizes of the formed complexes were ≈200 nm at N/P ratios of 0.5 and higher, while the surface charge of the complexes transitioned from negative (−20 mV) to positive (+44.2 mV) with an increasing N/P ratio (Figure S1b and Table S3, Supporting Information). The mobility of pDNA molecules in the agarose gel gradually decreased with increasing amounts of CTP up to an N/P ratio of 1.0 and was completely retarded at higher N/P ratios (2.5−25) (Figure S1c, Supporting Information). The analyses of free peptides in the pDNA/CTP complex solutions formed at N/P ratios = 0.5–5.0 revealed that up to 64% of CTP gradually bound to the pDNA molecules. However, this binding between pDNA and CTP is in pDNA concentration‐dependent manner as indicated by the excessive amount of free CTP in the complex solutions formed at higher N/P ratios (Figure S1d,e, Supporting Information). We subsequently examined the transfection efficiency of the pDNA/CTP complexes formed at various N/P ratios in intact *Arabidopsis thaliana* (*A. thaliana*) leaves, a model plant species harboring ≈100 copies of plastid per leaf cell. The transfection efficiency, represented by Rluc activity, was assayed using leaves sampled 24 h after infiltration. Maximum Rluc activity was observed in plant leaves transfected with pDNA/CTP complexes formed at an N/P ratio of 1.0, while a significant decrease in activity was seen in samples treated with complexes formulated at higher N/P ratios (Figure S1f, Supporting Information). Morphological studies by atomic force microscopy (AFM) revealed the interaction between pDNA molecules and KH_9_‐OEP34 at an N/P ratio of 0.1 and condensation of pDNA by KH_9_‐OEP34 to form globular‐shaped complexes at an N/P ratio of 0.5–1.0 (Figure S1g, Supporting Information).

A previous study showed that the incorporation of a CPP into a mitochondria‐targeted peptide/pDNA complex can significantly increase the mitochondria‐targeted transgene expression in *Arabidopsis* leaves.[qv: 12a] We coupled the pDNA/CTP complex with the CPP BP100 to improve the cell translocation efficiency of the peptide‐based complex for more efficient targeting of the plastids. The pDNA/CTP complexes prepared at an N/P ratio of 1.0 (resulting in the highest Rluc activity) were chosen to form the pDNA/CTP/CPP complexes at various BP100‐to‐pDNA ratios. The addition of BP100 to the pDNA/CTP complexes at N/P ratios of 0.1–10 gradually increased the complex sizes (ranging from 148 to 200 nm), while the surface charges of complexes transitioned to positive at an N/P ratio of 1.0 and at higher ratios (Figure 1b and Table S4, Supporting Information). The mobility of the pDNA molecule in the pDNA/CTP/CPP complexes gradually decreased with increasing N/P ratios before being completely retarded at an N/P ratio of 10 in the agarose gel matrix (Figure S2a, Supporting Information). Free peptide analyses indicated that up to 60% of BP100 gradually incorporated with the pDNA/CTP complexes to form pDNA/CTP/CPP complexes at N/P ratios = 0.1–2.5 (Figure S2b,c, Supporting Information). AFM imaging of the pDNA/CTP/CPP complexes formed at N/P ratios of 0.5, 1.0, and 2.5 showed a uniformed distribution of spherical complexes on the mica surface, indicating that there was no morphological alteration of the complex after the addition of BP100 (Figure 1c and Figure S2d,e, Supporting Information).

We then evaluated the transfection efficiency of these pDNA/CTP/CPP complexes by infiltrating the respective complex solutions into *A. thaliana* leaves and quantifying the transgene expression by the Rluc assay as before. A maximum enhancement in Rluc activity (2.1‐fold) was achieved using pDNA/CTP/CPP complexes formed at N/P ratios of 1.0 and 2.5 relative to that obtained by pDNA/CTP alone (Figure 1d). The addition of higher amounts of BP100 (N/P > 2.5) did not yield further improvement in the Rluc activity (Figure 1d). This result indicates that the incorporation of CPP (BP100) into the pDNA/CTP complexes significantly enhanced chloroplast‐targeted gene expression in intact plant leaves.

### 2.2 Optimization of the pDNA/CTP/CPP Complexes for Improved Gene Expression in Chloroplasts

BP100, the CPP used in this study, and its cationic domain‐conjugated derivatives, BP100‐KH_9_ and KH_9_‐BP100 (Table S1, Supporting Information), have demonstrated their abilities to function as gene carriers to deliver biomacromolecules into plant cells. These cell‐penetrating peptides were previously reported to infiltrate intact plants.^11^, ^12^ A separate study showed that the fusion of a cargo to the N or C‐terminal of BP100 influenced the efficiency of peptide delivery of the cargo to the target compartment within the plant cell.^13^ Therefore, we compared the transfection efficiency of pDNA/CTP in combination with BP100, BP100‐KH_9_, and KH_9_‐BP100 as CPPs. The morphology, size, and surface charge of clustered pDNA/CTP/CPP complexes formed with BP100‐KH_9_ or KH_9_‐BP100 at an N/P ratio of 1.0 were globular, relatively smaller (≈130 nm) than those formed using BP100, and positively charged (20 mV) (Figure S3a,b, Supporting Information). However, replacing the ternary complex with either BP100‐KH_9_ or KH_9_‐BP100 as CPP did not improve the chloroplast transfection efficiency in *A. thaliana* (Figure S3c, Supporting Information). These results suggest that BP100 is the most suitable for use as a CPP in the clustered pDNA/CTP/CPP complex.

**Figure 2:**
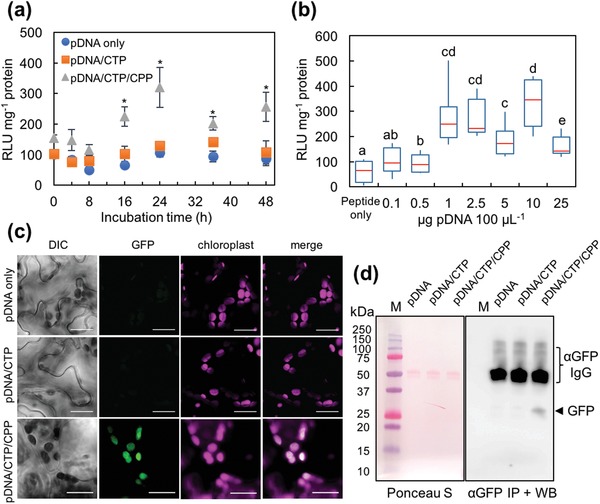
Expression profiles of reporter proteins in the chloroplasts of *A. thaliana* leaves delivered using clustered pDNA/CTP/CPP complexes. a) Time‐course analysis of *Renilla* luciferase activity at different time points after transformation. The pDNA is in the form of p*PpsbA::Rluc*. The pDNA/CTP (KH_9_‐OEP34) and pDNA/KH_9_‐OEP34/BP100 complexes were formed at an N/P ratio of 1.0 with a pDNA amount of 1.0 µg in 100 µL of transformation solution. Error bars represent the SD. Asterisks indicate a significant difference compared to samples infiltrated with pDNA only (Student's *t*‐test, *n* = 4, *p* < 0.01). b) *Renilla* luciferase activities in *Arabidopsis* leaves transformed with a clustered pDNA(p*PpsbA::Rluc*)/KH_9_‐OEP34/BP100 complex solution (N/P ratio = 1.0) with different amounts of pDNA in 100 µL of transformation solution. The data from eight biologically independent samples (*n* = 8) for each treatment are shown in the form of a box plot. Red lines represent the median value. Significant differences among treatments, indicated by different letters, were analyzed by one‐way ANOVA and Tukey's HSD test at *p* = 0.05. c) Transient expression of plastid‐specific GFP in *Arabidopsis* leaf cells, mediated by the clustered pDNA(p*Prrn::GFP(S65T)::TpsbA*)/KH_9_‐OEP34/BP100 complexes, was observed under a confocal laser scanning microscope at 24 h post‐transformation. The autofluorescence of chloroplasts is shown in magenta. Scale bars = 20 µm. d) Verification of plastid‐specific GFP expression in *Arabidopsis* chloroplasts by immunoprecipitation and immunoblotting. The image on the left shows the equal loading of immunoprecipitated proteins after transfer onto the PVDF membrane and staining with the Ponceau S solution. The image on the right shows the chemiluminescent signal of an HRP‐conjugated antibody against the anti‐GFP antibody used for the immunoblot analysis. Lanes indicated with the letter M are loaded with the dual‐stained Precision Plus protein marker (Bio‐Rad Laboratory). The arrow indicates the GFP (≈27 kDa) band after exposure to LAS3000 for 5 min.

We subsequently examined the expression profiles of pDNA encoding Rluc with the *psbA* promoter (p*PpsbA::Rluc*) in plastids 48 h post‐transfection with pDNA/KH_9_‐OEP34/BP100 complexes. Plants transfected with the clustered pDNA/CTP/CPP complexes exhibited the highest Rluc activity at 24 h after infiltration and slightly declined at 36–48 h post‐transfection (**Figure** **2**a). The effect of pDNA concentration on the transfection efficiency was then studied by infiltrating the leaves with clustered complexes formed at an N/P ratio of 1.0 at various pDNA concentrations. At 24 h post‐infiltration, leaves transformed using the clustered complexes with pDNA concentrations ranging from 1.0 to 10 µg per 100 µL exhibited significantly higher Rluc activity than leaves transformed with peptide only (1 µg peptide 100 µL^−1^) (Figure 2b). However, the Rluc activity decreased significantly in leaves transformed using the clustered complexes with a pDNA concentration of 25 µg per 100 µL.

To confirm the chloroplast‐targeted transgene expression mediated by clustered pDNA/CTP/CPP complexes, we employed the expression of a point mutation‐improved fluorescent variant of green fluorescent protein gene (*GFP(S65T)*)^14^ controlled by the plastid‐specific constitutive *rrn16* promoter (*Prrn*) and 3′‐untranslated regions of the *psbA* gene (*TpsbA*) in a transient expression vector p*Prrn::GFP(S65T)::TpsbA* (Figure S4a and Table S2, Supporting Information). The p*Prrn::GFP(S65T)::TpsbA*/KH_9_‐OEP34 complexes were formulated at N/P ratios = 0.1, 0.5, 1.0, 5.0, and 10 (Figure S4b, Supporting Information). Characterizations of the complex size, surface charge, and morphology of the pDNA/CTP and clustered pDNA/CTP/CPP complexes were performed previously. Incorporation of p*Prrn::GFP(S65T)::TpsbA* with the increasing amount of CTP gradually decreased the hydrodynamic diameter of pDNA/CTP complexes formed at N/P ratios up to 5.0 while the surface charges of these complexes were progressively increased (Figure S4b and Table S5, Supporting Information). Mobility of pDNA molecules in the electrostatic field was regularly retarded with increasing amount of CTP in the complex solutions (Figure S4c, Supporting Information). Based on our transfection efficiency in p*PpsbA::Rluc*/CTP complexes‐transfected *Arabidopsis* leaves, globular pDNA/CTP complexes with a particle size of 190 nm formed at an N/P ratio of 1.0 (Figure S4b–d, Supporting Information) were chosen to perform plastid‐specific GFP expression analysis by *Arabidopsis* leaf infiltration. However, leaves transformed with these complexes showed no GFP expression (Figure 2c). We then modified the pDNA/CTP complex by adding BP100 to form the clustered pDNA/CTP/CPP complex to enhance the cellular internalization of pDNA/CTP complexes. Increasing the amount of CPP gradually increased the particle sizes of pDNA/CTP/CPP complexes formed at different N/P ratios (Figure S5a and Table S6, Supporting Information). The surface charges of the pDNA/CTP/CPP complexes were progressively increased (Figure S5a, Supporting Information). The electrophoretic gel mobility‐shift assays showed that the pDNA molecules were slowly released from the pDNA/CTP/CPP complexes formed at higher N/P ratios when compared to pDNA/CTP complexes (Figure S5b, Supporting Information). Regarding this, the negatively charged, spherical, small complex (170 nm) of clustered pDNA/CTP/CPP with ≈40% of peptide incorporation at an N/P ratio of 1.0 (Figure S5c–e, Supporting Information) was chosen for expression analysis. At 24 h post‐transformation, the fluorescent signal of GFP was detected in the chloroplasts of *Arabidopsis* leaves (Figure 2c and Figure S6, Supporting Information). For further confirmation of GFP expression in the chloroplasts of transformed leaves, we isolated the chloroplasts from *Arabidopsis* leaves transformed with p*Prrn::GFP(S65T)::TpsbA* only, pDNA/CTP complexes, or clustered pDNA/CTP/CPP complexes, and immunoprecipitated the GFP from the chloroplast proteins for detection by immunoblot analysis. Western blotting of the immunoprecipitated fractions of *Arabidopsis* chloroplast proteins showed that GFP accumulated in the chloroplasts of leaves transformed with clustered pDNA/CTP/CPP complexes (Figure 2d). No GFP accumulation was detected in the immunoprecipitated fractions of *Arabidopsis* chloroplast proteins isolated from leaves transfected with pDNA only or with pDNA/CTP complexes. This result confirms that the clustered CTP/CPP peptide complexes serve as an effective carrier to enable transgene expression by chloroplasts in intact plants.

We further elucidated the involvement of CTP and CPP peptides in targeting the pDNA delivery to plastids by analyzing the transgene expression in pDNA/BP100 complexes‐transfected *Arabidopsis* leaves. The p*PpsbA::Rluc*/BP100 complexes were formed at different N/P ratios. These p*PpsbA::Rluc*/CPP complexes exhibited different physicochemical properties from that of pDNA/CTP and pDNA/CTP/CPP complexes (Figure S7a,b, Supporting Information). However, these pDNA/CPP complexes did not show significant transfection efficiency in plant leaves (Figure S7c, Supporting Information). Nonetheless, transfecting the *Arabidopsis* leaves with p*Prrn::GFP(S65T)::TpsbA*/CPP complexes showed no chloroplast‐specific GFP expression (Figure S7d,e, Supporting Information). These results indicate that the CTP KH_9_‐OEP34 plays a crucial role in the mixed CTP/CPP carrier system to target the gene delivery to plastids.

### 2.3 Expression of Reporter Genes in *Nicotiana benthamiana* Chloroplasts

**Figure 3:**
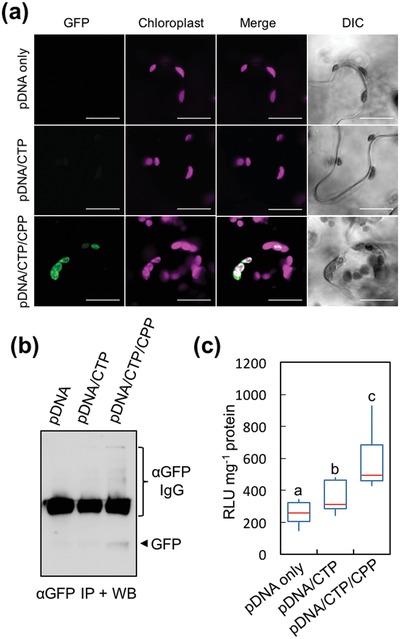
Transient expression of reporter proteins in the chloroplasts of *N. benthamiana* leaf cells mediated by the clustered pDNA/CTP/CPP complexes. a) CLSM observation of GFP in the chloroplasts at 24 h after infiltration with pDNA (p*Prrn::GFP(S65T)::TpsbA*) only, pDNA/KH_9_‐OEP34 complexes, or clustered pDNA/KH_9_‐OEP34/BP100 complexes. Scale bars = 20 µm. b) Immunoprecipitation and western blotting of GFP expressed in *N. benthamiana* leaf chloroplasts at 24 h post‐transformation with pDNA (p*Prrn*::*GFP(S65T)::TpsbA*) only, pDNA/KH_9_‐OEP34 complexes, or clustered pDNA/KH_9_‐OEP34/BP100 complexes. The arrow indicates the GFP band on the membrane after chemiluminescence detection for 5 min. c) *Renilla* luciferase activities in *N. benthamiana* leaves at 24 h after infiltration with p*PpsbA::Rluc* only, pDNA/KH_9_‐OEP34 complexes, and pDNA/KH_9_‐OEP34/BP100 complexes at an N/P ratio of 1.0. The distribution in luciferase activity of the eight leaf samples infiltrated with different solutions (*n* = 8) is represented in the form of a box plot. Red lines represent the median value. Different letters indicate significant differences among the mean luciferase activities analyzed by one‐way ANOVA and Tukey's HSD test at *p* = 0.05.

To test the ability of the clustered complexes to transfect the chloroplast in other plant species, we applied the most efficient chloroplast‐targeted peptide‐based formulation for the transient expression of transgene in intact *Nicotiana benthamiana* (*N. benthamiana*) leaves, the most widely used plant leaf materials in plant molecular biology. pDNA/CTP and clustered pDNA/CTP/CPP complexes were formulated using *Rluc* and *GFP* expression vectors at an N/P ratio of 1.0 and the previously optimized pDNA concentration (1.0 µg 100 µL^−1^). The chloroplast‐targeted transgene expression in *N. benthamiana* leaves mediated by the *GFP*‐encoding p*Prrn::GFP(S65T)::TpsbA*/CTP/CPP complex was verified by confocal laser scanning microscopic (CLSM) observation and immunoblotting. As with *A. thaliana*, we could not detect the GFP signal in the chloroplasts of leaves transformed with p*Prrn::GFP(S65T)::TpsbA* only or with pDNA/CTP complexes (**Figure** **3**a and Figure S8, Supporting Information). In contrast, we found a significant number of chloroplasts with distinct GFP signals within a single mesophyll cell of the plant transformed with the clustered pDNA/CTP/CPP complexes at 24 h post‐infiltration (Figure 3a and Figure S8, Supporting Information). After immunoprecipitation with anti‐GFP antibody and immunoblotting, we detected the GFP‐specific band only in the immunoprecipitated protein fraction obtained from chloroplasts isolated from leaves transformed using clustered pDNA/CTP/CPP complexes (Figure 3b and Figure S9, Supporting Information). Compared to p*PpsbA::Rluc*‐only treatment, *N. benthamiana* leaves transformed using p*PpsbA::Rluc*/CTP complexes showed a 1.4‐fold increase in Rluc activity, whereas the Rluc activity in leaves transformed using clustered pDNA/CTP/CPP complexes was notably higher (2.2‐fold) (Figure 3c). These results imply that the clustered CTP/CPP peptide‐based gene carrier can be effectively used independently of plant types to mediate transgene expression in chloroplasts.

### 2.4 Clustered pDNA/CTP/CPP Complex‐Mediated Transformation of Nongreen Plastids

**Figure 4:**
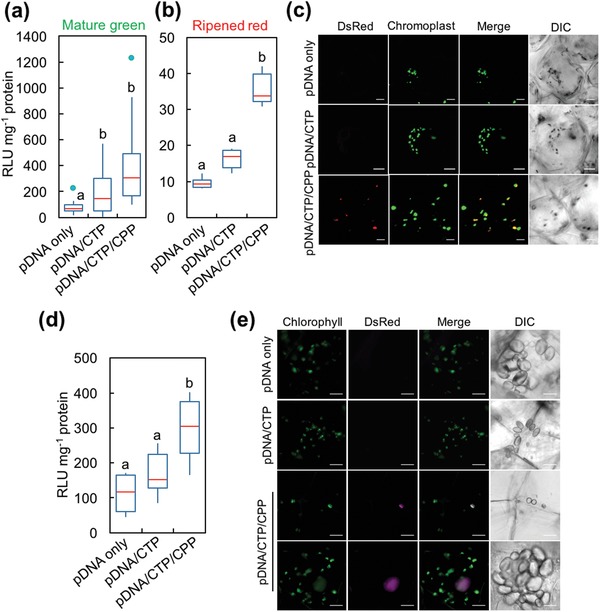
Expression of reporter proteins in nongreen plastids of tomato and potato mediated by the clustered pDNA/CTP/CPP complexes. a,b) *Renilla* luciferase activities in two different stages of tomato fruit at 24 h after infiltration with p*PpsbA::Rluc* only, pDNA/KH_9_‐OEP34 complexes, and clustered pDNA/KH_9_‐OEP34/BP100 complexes at an N/P ratio of 1.0. Tomato fruits were injected with 100 µL of complex solution with a needle‐attached syringe. The distribution of luciferase activities in 12 mature‐green fruits and 6 ripened‐red fruits for each treatment (*n* = 12 and *n* = 6, respectively) is shown in the form of a box plot. Red lines indicate the median value. The green dots in (a) represent the outliers. Different letters indicate significant differences between the groups of data analyzed by one‐way ANOVA and Tukey's HSD test at *p* = 0.05. c) Expression of DsRed in the chromoplasts of mesocarpic cells in the ripened‐red fruit of the tomato. Scale bars = 20 µm. d) Rluc activities in the potato tuber slices transformed with pDNA (p*PpsbA::Rluc*) only, pDNA/KH_9_‐OEP34 complexes, and clustered pDNA/KH_9_‐OEP34/BP100 complexes at an N/P ratio of 1.0. Different letters indicate significant differences between treatments analyzed by one‐way ANOVA and Tukey's HSD test at *p* = 0.05 (*n* = 8). e) CLSM observation of DsRed expression mediated by clustered p*Prrn::DsRed::TpsbA*/CTP/CPP complexes in the amyloplast of a potato tuber at 24 h after vacuum infiltration. Scale bars = 10 µm.

Nongreen plastids such as chromoplasts, leucoplasts, and amyloplasts have been transplastomically engineered to enhance the production of health‐promoting compounds such as vitamin A and commercially important biopolymers such as polyhydroxyalkanoates.^15^ To enable transgene expression in these plastids, we employed the clustered pDNA/CTP/CPP complexes for plastid transformation of two plants in the Solanaceae family, namely, tomato and potato. Tomato has been shown to differentiate chloroplasts into carotenoid‐accumulating chromoplasts during the transition of the mature‐green fruit to the ripened‐red fruit.[qv: 3b,16] First, we evaluated the efficiency of targeting transgene (*Rluc*) expression to plastids by introducing clustered p*PpsbA::Rluc*/CTP/CPP complexes into mature‐green and ripened‐red tomato fruit via syringe injection (Figure S10, Supporting Information). At the mature‐green stage, fruit transformed with the pDNA/CTP complexes showed a significant, 2.3‐fold higher Rluc activity than that of the fruit injected with pDNA only (**Figure** **4**a). Notably, modifying the surface of the p*PpsbA::Rluc*/KH_9_‐OEP34 complexes with BP100 resulted in a fivefold higher Rluc activity (relative to the basal Rluc activity in the pDNA‐only control fruit) in the mature‐green fruit (Figure 4a). The basal Rluc activity in ripened‐red fruit injected with p*PpsbA::Rluc* only was distinctly lower than that in the mature‐green fruit (Figure 4a,b). Similarly, ripened‐red fruit injected with pDNA/CTP/CPP complexes displayed an ≈3.5‐fold higher Rluc activity than that in fruit injected with pDNA only (Figure 4b). These results imply that the clustered pDNA/CTP/CPP complexes can mediate the transformation of different types of plastids in the two different developmental stages of the tomato fruit.

To confirm the chromoplast‐specific expression of a transgene delivered by the clustered peptide complexes, we constructed a plasmid encoding red fluorescent protein (DsRed) and a plastid‐specific promoter, which can distinguish the autofluorescence of the carotenoids in the chromoplast of the ripened‐red fruit. The expression of the *DsRed* gene was transcriptionally controlled by the constitutive, plastid‐specific *rrn16* promoter and *psbA* terminator (Figure S11a and Table S2, Supporting Information). Characterizations of the p*Prrn::DsRed::TpsbA*/CTP and pDNA/CTP/CPP complexes were carried out as previously performed. The hydrodynamic diameters of pDNA/CTP complexes formed at various N/P ratios up to 5.0 steadily decreased (Figure S11b and Table S7, Supporting Information). The surface charges of pDNA/CTP complexes significantly increased at N/P ratios = 1.0–10 (Figure S11b, Supporting Information). Gel retardation assays showed that the mobility of pDNA molecules in the electrostatic field was gradually retarded with increasing N/P ratios (Figure S11c, Supporting Information). The pDNA/CTP complexes formed at an N/P ratio of 1.0 were small, globular (Figure S11d, Supporting Information), negatively charged, and hence selected for surface modification with BP100. CPP BP100 was successfully incorporated into the pDNA/CTP complexes, indicated by the reduction of hydrodynamic diameters and increase of the surface charges of pDNA/CTP/CPP complexes formed at different N/P ratios (Figure S12a and Table S8, Supporting Information). The electrophoretic mobility‐shift assays showed the retardation of pDNA molecules in the complex solutions formed at higher N/P ratios (Figure S12b, Supporting Information). The resulting pDNA/CTP/CPP complexes with the particle sizes smaller than 115 nm and a negatively charged surface with ≈44% of CPP incorporation at an N/P ratio of 1.0 (Figure S12c–e, Supporting Information) were chosen for the transformation of ripened‐red tomato fruits.

Chromoplasts in the ripened‐red fruit can vary in appearance, ranging from globular to irregular structures or appearing as needle‐shaped crystalloid structures.[qv: 4b] Expression of DsRed in the chromoplast of the ripened‐red fruit was observed using CLSM imaging. We could not detect any DsRed signal in the chromoplast of the fruit at 24 h after injection with both the p*Prrn::DsRed::TpsbA*‐only control and pDNA/CTP complex solutions (Figure 4c, top and middle panels, and Figure S13, Supporting Information). In the fruit injected with the clustered pDNA/CTP/CPP complexes, however, clear DsRed signals that colocalized with the carotenoid autofluorescence in the chromoplasts could be seen at 24 h post‐infiltration (Figure 4c, bottom panel, and Figure S13, Supporting Information). This finding suggests that the CTP/CPP complexes can function as effective gene carriers to specifically target transgene expression to the chromoplast in tomato fruit.

In other sink organs such as the root and the tuber, the plant cell differentiates proplastids into noncolored plastids such as the leucoplast and a starch‐storage plastid called the amyloplast.^17^ Using our system, we attempted to transform these plastids into the root cell of the tomato. The root segments from tomato seedlings were vacuum infiltrated with either p*PpsbA::Rluc* only or pDNA/peptide complex solutions. No discernible differences in Rluc activity were seen in root segments transformed with p*PpsbA::Rluc* with or without CTP (Figure S14a, Supporting Information). Moreover, clustered pDNA/CTP/CPP complexes enhanced the Rluc activity in root segments by ≈2.0‐fold at 24 h after vacuum infiltration. Clustered p*Prrn::DsRed::TpsbA*/CTP/CPP complexes were introduced into the tomato root to confirm the plastid‐specific transgene expression in the root cells by CLSM imaging. While the root cells transformed with only p*Prrn::DsRed::TpsbA* did not show any DsRed fluorescence, a plastid‐localized DsRed signal was observed in root cells transformed with clustered pDNA/CTP/CPP complexes at 24 h post‐transformation (Figure S14b,c, Supporting Information).

We subsequently extended the application of our system by delivering pDNA using the clustered peptide‐based carriers into the amyloplasts of potato tubers. Potato slices transformed with the p*PpsbA::Rluc*/CTP complexes, assayed after 24 h of transformation, exhibited Rluc activity at levels comparable to that of the pDNA‐only control (Figure 4d). In contrast, potato slices transformed with p*PpsbA::Rluc*/CTP/CPP showed significantly higher (2.7‐fold increase compared to pDNA‐only treatment) levels of Rluc activity (Figure 4d). We also checked the specificity of transgene expression in the amyloplast by CLSM observation. At 24 h after infiltration of the potato slices with the clustered p*Prrn::DsRed::TpsbA*/CTP/CPP complexes, we observed the increasing fluorescent signals arising from DsRed overlapping with the amyloplast (chlorophyll autofluorescence was induced by exposure of potato tuber slices to light) (Figure 4e and Figure S15, Supporting Information). In sum, we concluded that the clustered pDNA/CTP/CPP complexes improved the transformation efficiency of storage plastids in the sink cells of tomato roots and potato tubers by enhancing the ability of the cargo to penetrate the cellular barriers.

### 2.5 Subcellular Distribution and Targeting of pDNA to Plastids Mediated by Clustered CTP/CPP Carriers

**Figure 5:**
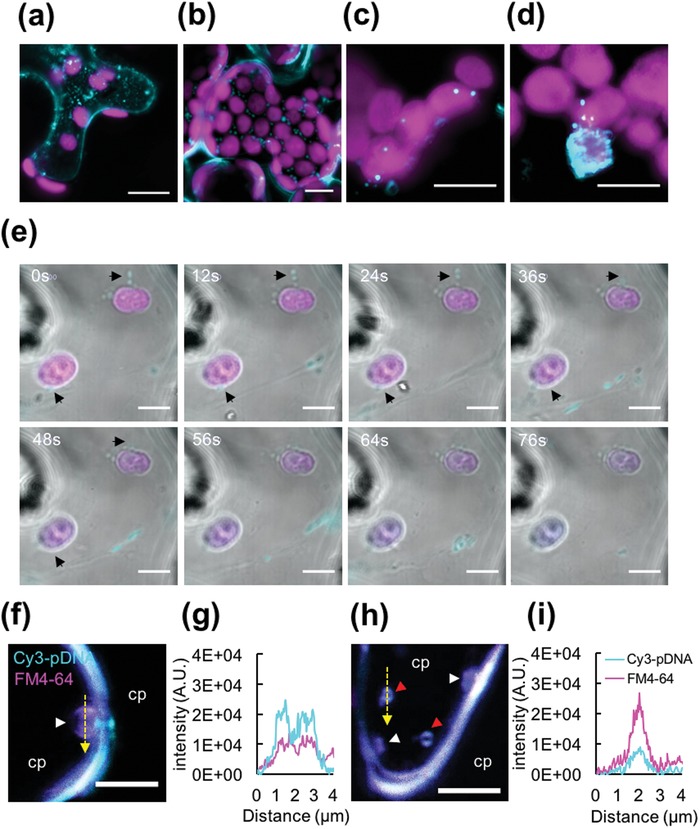
Targeting pDNA delivery to the plastid in a plant cell using a combination of a CTP and CPP. a,b) Internalization of the clustered pDNA/CTP/CPP complexes into plant cells. CLSM images of an *N. benthamiana* leaf at 2 h after transformation with Cy3‐labeled pDNA/CTP/CPP complexes. CLSM observation of a) the spongy mesophyll cells and b) palisade mesophyll cells reveals the presence of fluorescent Cy3‐labeled pDNA molecules (cyan) delivered using a CTP/CPP complex into cells. Autofluorescent signals of chlorophyll are shown in magenta. c,d) Targeting of pDNA molecules to the plastids. c) At 2 h post‐infiltration, the Cy3‐labeled pDNA/CTP/CPP complexes (cyan) were found to colocalize with the plastids (magenta) in the plant cells. d) One of several plastids extensively covered by the Cy3‐labeled pDNA/CTP/CPP complexes. e) Time‐course observation of Cy3‐labeled pDNA/CTP/CPP complexes delivered to the chloroplasts. Arrowheads indicate the original positions of the complexes before approaching the chloroplasts. f–i) Vesicle formation in the Cy3‐pDNA/CTP/CPP complex‐transfected cells. Colocalization of the Cy3‐pDNA/CTP/CPP complexes (cyan) with the plasma membrane‐staining fluorescent dye FM4‐64 (magenta) observed by CLSM imaging at 2 h post‐infiltration. The white arrowhead indicates the membrane‐bound vesicles in (f) and (h), while the red arrowheads indicate the free vesicles in the cytoplasm (cp) in (h). g,i) Fluorescent profiles (represented by the yellow dashed line across the vesicles) indicate the colocalization of the clustered pDNA/CTP/CPP complexes with membrane‐bound vesicles in (f) or free vesicles in (h). The plant cell membrane was stained with FM4‐64 (magenta). Scale bars = 10 µm in (a) and (b), 5 µm in (c)–(e), and 4 µm in (f) and (h).

Intracellular distribution studies using CLSM observations of clustered cyanine‐3 (Cy3) labeled pDNA/CTP/CPP complexes in plant cells revealed that the Cy3‐labeled pDNA molecules were predominantly localized in the cytoplasm of transformed plant cells after 2 h incubation (**Figure** **5**a,b). We detected Cy3 fluorescence in multiple chloroplasts within the plant cells (Figure 5c), with selected chloroplasts extensively covered by Cy3 fluorescence (Figure 5d). Time‐course observation of the pDNA/peptide complexes in *N. benthamiana* leaf cells revealed that the complex was directly transported to the chloroplasts via the intracellular network and merged with the chloroplast membranes at 2 h post‐infiltration (Figure 5e and Movie S1, Supporting Information). *Z*‐stack imaging of chloroplasts in plant cells transformed with Cy3‐labeled pDNA/CTP/CPP complexes showed that the complexes interacted with the chloroplast membrane and were internalized into the chloroplast to release the pDNA cargo (Figure S16, Supporting Information).

We then followed the internalization of the clustered pDNA/CTP/CPP complexes through the plant cell boundaries by colocalization with the plasma membrane‐staining fluorescent dye, FM4‐64. Incorporation of FM4‐64 into the complex solution did not interfere with the properties of the clustered Cy3‐labeled pDNA/CTP/CPP complexes (Figure S17, Supporting Information). We found that the clustered Cy3‐labeled pDNA/CTP/CPP complexes colocalized with the large (≈2 µm), plasma membrane‐bound vesicles (stained with FM4‐64) in plant cells infiltrated with the Cy3‐labeled pDNA/CTP/CPP complexes after 2 h of incubation (Figure 5f–i). These vesicles decreased (≈0.5 µm) after detaching from the plasma membrane and were trafficked as free vesicles in the cytoplasm (Figure 5h,i). Altogether, these results indicated that the clustered pDNA/CTP/CPP cargos were able to enter the plant cells by inducing the formation of large vesicles, following which the pDNA molecules were transported to the plastids by endomembrane trafficking on the intracellular network, where the pDNA molecules were finally released.

## 3 Discussion

Electrostatic interaction between the negatively charged pDNA molecules and the positively charged cationic chloroplast‐targeting peptide KH_9_‐OEP34 enables the condensation of pDNA to form spherical pDNA/CTP complexes. The surface charges of the complex transition from negative to positive with an increasing N/P ratio. This peptide/DNA complexation is a kinetically and thermodynamically driven process that may cause the variation in physicochemical properties and morphologies of different pDNA/peptide complexes.^18^ Interaction of the pDNA molecule with the peptide has been also shown to modulate the secondary structure of the CTP, decreasing its ability to deliver pDNA molecules to plastids,^19^ particularly at higher N/P ratios.[qv: 12a,20] However, incorporating the different lengths of pDNA molecules with CTP/CPP results in distinctive pDNA/peptide complexes with miscellaneous physicochemical characteristics, but does not significantly affect the transfection efficiency.^21^ In addition, the decrease in transformation efficiency of the pDNA/CTP complexes formed at higher N/P ratios, above the optimal point, may be due to the slow dissociation of pDNA/CTP complexes once delivered into the plastids.^22^ In contrast, the pDNA/CTP complexes formed at suboptimal/lower N/P ratios may destabilize prematurely and release the bound pDNA before reaching the plastids via the membrane‐associated organelle uptake of pDNA/CTP complexes, as shown in a previous report.[qv: 12b]

The addition of positively charged, membrane‐binding, cationic CPP BP100 to the pDNA/CTP complexes resulted in the sub‐micrometer‐sized clustered pDNA/CTP/CPP complexes that enhanced the plastid transfection efficiency. Furthermore, we found that complexes formed using BP100, BP100‐KH_9_, or KH_9_‐BP100 exhibited similar sizes but different transfection efficiencies. Higher transgene expression levels were achieved using free BP100, revealing the crucial role of the CPP in inducing the uptake of the clustered pDNA/CTP/CPP complexes into the plant cell, whereby it should be freely exposed on the surface of the complex.^13^ Our cellular internalization experiment showed that the clustered pDNA/CTP/CPP complexes are preferably internalized into plant cells by inducing large intracellular vesicle formation and trafficking. Nanoparticles with sizes ranging from 120 to 200 nm are principally suitable for cellular uptake by plant cells.^23^ These complexes can translocate across the plant cell boundaries to the plastids after dissociating from the large endosomes.^24^ The presence of BP100 on the surface of the complexes may also, as an added advantage, contribute to resistance against degradation by foreign molecules in the intracellular space prior to the disintegration of DNA/CTP complexes from the CPP for DNA release in the plastid.^25^

The efficiency of CTP in combination with CPP to deliver the pDNA molecule across the plasma membrane and subsequently across the plastid membrane bilayer is relatively similar among the differentiated types of plastids in various cell types. It is well known that there are up to a hundred chloroplasts in one leaf mesophyll cell^1^, ^26^ whereas there are ≈70 plastids in one tomato fruit pericarpic cell.[qv: 3b,27] Interestingly, we found that the leaves of *A. thaliana* and *N. benthamiana* as well as mature‐green tomato fruits, regardless of cell type, can be transformed by the clustered pDNA/CTP/CPP complexes with similar efficiencies. Furthermore, our transgene expression results in the chromoplasts of the ripened‐red tomato fruit, leucoplasts and amyloplasts of the tomato root, and the potato tuber show that the CTP/CPP complex is capable of transforming even nongreen plastids, albeit at a lower rate. This finding indicates that transient gene expression in plastid using peptide‐based carriers is not dependent on plastid membrane topologies or the number of plastids per cell. The overall transcription and translation rates of plastid genes involved in photosynthesis during the transition of chloroplast to chromoplast or chloroplast to amyloplast were found to be significantly downregulated in tomato fruits and potato tubers.[qv: 3a,28] Since the promoter used to drive *Rluc* gene expression in our quantitative studies is the light‐inducible *psbA* promoter, the transgene expression level in nongreen plastids would be lower than that in the chloroplasts.^29^ Instead, the nongreen plastid‐targeted gene expression mediated by the clustered CTP/CPP complexes can be further improved by employing chromoplast/amyloplast‐specific promoters or the constitutive *rrn16* promoter that functions in various types of plastids for the regulation of gene expression.[qv: 3a,7a,29a]

Gene transfer to plastids in both model plants (*A. thaliana* and *N. benthamiana*) and crop species (*Solanum lycopersicum* (*S. lycopersicum*) and *Solanum tuberosum*) through conventional techniques is technically challenging while it is a tranquil routine in *Nicotiana tabacum* (*N. tabacum*). The factors like specialized plant cell wall composition, tissue toughness, cytotoxicity of carrier to the plant cells, plastid density, and different plant regeneration rate govern the transformation efficiency in these species. Thus far, plastid transformation in photosynthetic and nonphotosynthetic tissues has been achieved via microprojectile bombardment,[qv: 5a,6,7] PEG‐mediated protoplast transformation,^8^ or precise microinjection of the DNA molecule into individual chloroplasts in the plant cell.^9^ Usually, a genetically stable transplastomic line can be achieved in 8–12 months after transformation using the conventional plastid transformation protocols.[qv: 5b] However, in the transient gene expression experiments, a single GFP‐positive plastid per cell in one treatment was considered sufficient for analysis, based on plastid transformation of *A. thaliana* and *N. tabacum* leaves, potato tuber slices as well as Marigold petals by particle bombardment and microinjection.[qv: 7a,9] As a comparative control experiment, we performed particle bombardment to elucidate the plastid transfection efficiency of this technique. Particle bombardment of p*PpsbA*::*Rluc*‐coated gold particles to the *Arabidopsis* leaves and two different stages of tomato fruit slices showed approximately twofold increases of Rluc activity compared to that in plant samples transfected by gold particle only (Figures S18a and S19a,b, Supporting Information). Transient expression of fluorescent proteins in the different types of plastids mediated by particle bombardment also provided relatively similar patterns as using the clustered CTP/CPP carrier system (Figures S18b and S19c,d, Supporting Information). However, transfecting the plant cells with particle bombardment might lead to the mislocalization of gene expression cassette since the gold particles are randomly projected into all compartments in the transfected plant cells (Figure S18c, Supporting Information).^30^ Using similar transient expression parameters (including the incubation time and pDNA amount per treatment), our mixed peptide‐based plastid transfection system showed higher efficiency and specificity to target the DNA delivery to plastids. We found ≈10−12 chloroplasts in *A. thaliana* or *N. benthamiana* leaves and five to six nongreen plastids in tomato fruit or potato slices to be positive for fluorescent protein expression at 24 h post‐infiltration with the clustered pDNA/CTP/CPP complexes. Moreover, *Renilla* luciferase activities in the various transformed plant tissues were at levels sufficiently high (3–10‐fold) for quantitative analysis at 16–48 h post‐transformation, indicating multiple successful transformed plastids per treatment. These expression levels might substantially alter the function of the plastid gene that requires marginal change to elevate its biological role in the plant tissue.[qv: 3a] This result suggests that our mixed peptides carrier system overcomes the current limitations of plastid transformation. This could be due to the enhanced abilities of the clustered CTP/CPP complexes in translocating and targeting the pDNA molecules into the plastids. Nevertheless, our current clustered peptides‐based gene delivery shortens the experimental time required for comprehensive study of biomolecules in a particular type of plastids.

Nanocarriers are talented materials for the gene delivery to plant cells but lack the activity to specify biomolecule delivery to the target plant organelles. Interestingly, there is a very recent successful example of transporting the gene expression cassettes into the plastids using the chemically modified single‐walled carbon nanotubes.^31^ The most astounding ability of the clustered CTP/CPP peptide carrier is that it specifically targets the passive delivery of DNA molecules to the plastids by recognizing the membrane topologies with a specific CTP sequence. The peptide‐based carrier also potentially provides the physical barrier and protects the DNA molecule from the cellular nucleases by condensing the DNA to the core of DNA/peptide complex without influencing its function. Our peptide‐based transient expression technique enables the cutting‐edge technique for rapid *in‐planta* assays of plastid genes with unknown functions or lethal genes encoded by the plastomes. However, the public concerns regarding the use of exogenous DNA molecules to modify the plant genetic background constrain the DNA‐based plastome modification platforms. The peptide carrier is able to transport not only the DNA molecule but also biomacromolecule such as short‐RNAs, proteins, and complexes for understanding their imperative function in plastids. This clustered CTP/CPP carrier allows the multiple biomacromolecules delivery to plastids for efficient modification of metabolic process in these organelles. Although the selected CPP and CTP are able to translocate biomolecules across cellular and plastid membrane, there may be other superior CPP/CTP sequences and peptide combinations that enable higher transfection efficiencies. Fundamentally, the cell‐penetrating ability of the CPP and the plastid‐targeting ability of the CTP can be further improved by exploring new combinations of CTP and CPP based on currently known CPPs and CTPs. The BP100 and KH_9_‐OEP34 sequences can be further chemically modified to enhance the cell‐internalization and plastid‐translocation efficiencies of the peptides.^32^ Moreover, the biomacromolecules can also be efficiently transported to the other plant organelles like mitochondria and peroxisomes by substituting the CTP in the ternary complexes with organelle‐specific transporting peptides.[qv: 12a]

The petite CTP/CPP carrier has a remarkable ability to transverse across the tough plant cell wall and membranes to specifically deliver biomolecules to the plastids. Moreover, it can be employed to carry multiple regulatory biomolecules to control the metabolic fluxes of the commercially important plant compounds synthesized inside the plastids. The peptide‐based biomolecule delivery platform supports the development of high‐throughput technology and can enforce the quality trait improvement, animal and human food production, and bioactive compound manufacturing in the next‐generation smart farming. However, the major production cost of the peptide‐based organelle transformation platform currently relies on the syntheses of peptides and corresponding biomacromolecules. Developing the fabrication method and purification process of these components using the hybrid‐chemical syntheses^33^ and recombinant DNA technology may be the greatest solution to minimize the manufacturing costs of this outstanding technology.

## 4 Conclusion

Our current study presents the idea of using an organelle‐targeting peptide, i.e., a peptide with the ability to target DNA cargo to a precise intracellular compartment for plant organellome modification. Furthermore, the combination of a CTP and a CPP may provide a strategy to transport not only DNA but also other biomacromolecules (mRNA, protein, and complexes) and bioactive compounds to the plastids in any intact plant. Our clustered CTP/CPP complexes provide a new and simple yet effective tool for the functional analysis of genes and plastome engineering in various types of plastids of different intact plant tissues without the need for laborious material preparation or costly instruments. This advanced scientific platform provides a robust strategy that enables the manipulation of metabolic pathways inside the plastids and, more importantly, can alleviate public concerns regarding the safety of using transgenic materials due to the ability of the platform to modify plants nontransplastomically.

## 5 Experimental Section

*Plant Materials and Cultivation Conditions*: Seeds of *A. thaliana* col‐0 were germinated in soil (Promix, Rivière‐du‐Loup, Canada) supplemented with vermiculite at a ratio of 2:1 and incubated at 22 °C with 16/8 h light/dark periods at 100 µmol photons m^−2^ s^−1^ in a growth chamber for 4 d. The plants were then cultivated at 22 °C with 8/16 h light/dark periods at 80 µmol photons m^−2^ s^−1^ in the growth chamber. Fully expanded leaves of 3−4 week old plants were used in the experiments.

*N. benthamiana* and tomato (*S. lycopersicum cuv*. MicroTom) seeds were sown and germinated in soil at 25 °C with 12/12 h light/dark periods at 80 µmol photons m^−2^ s^−1^ in the growth chamber for 7 d. The plants were then cultivated in soil at 22 °C with 8/16 h light/dark periods at 80 µmol photons m^−2^ s^−1^ in the growth chamber. Only the third to fifth fully expanded leaves of 4−5 week old plants were used in the experiments. Mature‐green and ripened‐red tomato fruits were transformed with the complex solution. For tomato root transformation, seedlings were germinated on ½ Gamborg's B5 media supplemented with 2% sucrose and 0.7% agar at 25 °C with 12/12 h light/dark periods at 80 µmol photons m^−2^ s^−1^ in the growth chamber before vacuum infiltration.

Potato tubers were purchased from the local market. The tubers were washed with running tap water accompanied by gentle scrubbing of the skin before cutting them into 1 cm^3^ cubes. Potato slices were prepared using a razor with dimensions of 0.5 cm × 0.5 cm × 2 mm and presoaked in ½ MS media (pH 5.7) supplemented with 1% sucrose prior to transformation with the complex solution.

*Construction of Plasmid DNA*: To construct the p*PpsbA::Rluc* expression vector (Figure S1a and Table S2, Supporting Information), a *N. tabacum psbA* (*NtPsbA*) promoter fragment was cloned from the DNA extracted from *N. tabacum* leaves with the primer pair described in Table S9 (Supporting Information). The *Rluc* gene was amplified from the available pDONR‐*cox2::rluc* vector.[qv: 12a] The *NtPsbA* promoter fragment was subcloned into the *Xba*I/*Bam*HI restriction enzyme digestion sites, and the *Rluc* gene fragment was inserted into the *Bam*HI/*Eco*RI restriction digestion sites in the pMDC107 plasmid (TAIR accession: 1009003751). For the construction of p*Prrn::GFP(S65T)::TpsbA* and p*Prrn::DsRed::TpsbA* expression vectors (Table S2, Supporting Information), the *rrn* promoter fragment was amplified from the tobacco genome with the promoter‐specific primers described in Table S9 (Supporting Information). The *PsbA 3′‐UTR* fragment (*TpsbA*) was cloned from DNA extracted from the leaves of *N. tabacum*. The *rrn* promoter fragment and *GFP(S65T)* or *DsRed* fragments were subcloned into *Xba*I/*Bam*HI restriction enzyme digestion sites on the pUC19 vector (New England Biolabs, Ipswich, MA, USA), and the *TpsbA* fragment was inserted into the *Bam*HI/*Eco*RI restriction sites of the resultant plasmids. Plasmid DNA molecules used in the transformation experiments were extracted from the liquid culture of *Escherichia coli* using a QIAGEN Plasmid Giga kit according to the manufacturer's protocol (QIAGEN, Hilden, Germany). Purified plasmid DNA was kept at −20 °C and a concentration of 1.0 mg pDNA mL^−1^.

*Cell‐Penetrating Peptides and Chloroplast‐Targeting Peptide*: The CTP KH_9_‐OEP34 and CPPs BP100, BP100‐KH_9_, and KH_9_‐BP100 were synthesized as previously described.[qv: 11a,b,12b] Their molecular weights were confirmed by matrix‐assisted laser desorption/ionization time‐of‐flight. Isocratic points and net charges at pH 7.0 were computed based on their sequences as shown in Table S1 (Supporting Information).

*Formation and Characterization of the Clustered Plasmid DNA/Peptide Complexes*: The pDNA/peptide complexes were formulated based on the N/P ratio, the ratio of moles of amine groups (NH_3_^+^) in the CTP and CPP to the negatively charged groups (PO_4_^−^) in the pDNA molecules.^34^ Plasmid DNA/KH_9_‐OEP34 (pDNA/CTP) complexes were formed at different N/P ratios by adding different amounts of KH_9_‐OEP34 peptide solution (1.0 mg mL^−1^ stock solution) to 10 µg of pDNA in water to a final volume of 100 µL. The solutions were mixed by vortexing and incubated at 25 °C for 30 min without shaking. After incubation, different amounts of cell‐penetrating peptide BP100 (1.0 mg mL^−1^ in water) were added to the pDNA/CTP complex solution to form the pDNA/CTP/CPP complexes at different N/P ratios. The solutions were mixed by vortexing and incubated at 25 °C for 30 min without shaking. The pDNA/KH_9_‐OEP34 or pDNA/KH_9_‐OEP34/BP100 complex solutions were then diluted with 700 µL of water. The particle size of the complexes was determined by dynamic light scattering, and the polydispersity index was determined with a Zeta Nanosizer using a 633 nm He‐Ne laser at 25 °C with a backscatter detection angle of 173°. The surface charge of the complexes was measured using a zeta potentiometer. Averaged data from three replicates were obtained using Zetasizer software version 6.20 (Malvern Instruments, Ltd., Worcestershire, UK). Electrostatic mobility shifts and release of the pDNA molecule from the complexes were analyzed by resolving 20 µL of complex solution in an 0.8% w/v agarose gel in 1 × tris‐acetate‐EDTA (TAE) buffer at 100 V for 30 min. Formation of complexes comprising pDNA/CTP and either N or C‐terminal cationic domain fused BP100 derivatives (KH_9_‐BP100 and BP100‐KH_9_) was carried out at an N/P ratio of 1.0 (CPP to pDNA), and the characterizations were performed as described above.

Morphologies of the pDNA/peptide complexes were examined by tapping mode AFM. Twenty microliters of complex solution prepared at an N/P ratio of 1.0 was spotted onto the freshly cleaved surface of mica and air dried at 25 °C for 3 h. The samples were then rinsed thoroughly with water and air dried in a desiccator (25 °C, 20% relative humidity) for 6 h to overnight. Complexes were observed in air at room temperature using a silicon cantilever (SI‐DF3, Hitachi High‐Tech Science Corporation, Tokyo, Japan) with a spring constant of 1.7 N m^−1^ in tapping mode AFM5300E (Hitachi High‐Tech Science Cooperation).

*Free Peptide Analyses*: The pDNA/CTP and pDNA/CTP/CPP complexes were formed at different N/P ratios. The complex solutions and the solutions containing peptide without the pDNA molecules were filtered through 0.1 µm Ultrafree‐MC centrifugal filter units (Merck KGaA, Darmstadt, Germany) to separate free peptides from the pDNA/peptide complexes. Total peptide amount in the solution was determined by BCA Protein Assay Kit (Pierce Biotechnology).

Purity of the free peptides isolated from the pDNA/CTP and pDNA/CTP/CPP complexes was confirmed by the reverse‐phase high‐performance liquid chromatography. The system composed an auto sampler (AS‐2055, JASCO, Tokyo, Japan), gradient pump (PU2089, JASCO), column oven (CO‐4060, JASCO), and C18 column (YMC‐Triart C18, particle size 5 µm, 150 × 4.6 mm i.d., YMC, Kyoto, Japan) with a 210 nm UV detector. The gradient mode of mobile phase consisted of 10%–55% acetonitrile + 0.1% trifluoroacetic acid was used to analyze the free peptide over 30 min with the flow rate of 1.0 mL min^−1^.

*Introduction of Clustered Complexes into Different Plant Tissues*: For leaf infiltration, ≈100 µL of complex solution containing 1.0 µg of plasmid DNA was introduced into the fully expanded leaves of *A. thaliana* and *N. benthamiana* from the abaxial side of the leaf using a 1 mL syringe without a needle. The infiltrated plant leaves were incubated under standard culture conditions for up to 48 h for expression analyses.

For root infiltration, ≈2 cm segments of the primary root from tomato seedlings were excised and rinsed twice with ½ Gamborg's B5 media supplemented with 2% sucrose without agar and then twice with water. Root segments were then placed into 500 µL of complex solution containing 1.0 µg plasmid DNA 100 µL^−1^, and the solution was vacuum infiltrated into the plant tissue at 600 mmHg for 5 min. After infiltration, the root segments were incubated in the complex solution for 30 min before three washes with ½ Gamborg's B5 media supplemented with 2% sucrose without agar. Transformed root segments were incubated in ½ Gamborg's B5 media supplemented with 2% sucrose with 0.7% agar in the dark at 22 °C for 24 h.

Intact mature‐green or ripened‐red tomato fruits (1.5−2.0 cm in diameter) were injected with 200 µL of complex solution containing 2.0 µg plasmid DNA using a 1 mL syringe with a 27 gauge needle attached. Two to three millimeters of the needle tip was gently inserted into the pericarp layer of the tomato fruit, and the solution was slowly injected into the cell layer. Only successfully infiltrated fruits, i.e., those that did not burst or have excess amounts of solution coming out from the fruit sepals (Figure S7, Supporting Information), were incubated under standard culture conditions for 24 h prior to the assessment of reporter gene expression.

Three to five potato tuber slices were preincubated in ½ MS + 0.7% agar supplemented with 1% sucrose at 22 °C in the dark at least 1 h after cutting. The potato slices were placed into 500 µL of complex solution (1.0 µg pDNA 100 µL^−1^), and the solution was vacuum infiltrated at 600 mmHg for 5 min. Following infiltration, the potato slices were left in the solution for 30 min before washing with ½ MS media + 1% sucrose without agar and then in ½ MS + supplemented with 1% sucrose and 0.7% agar. The transformed explants were incubated at 22 °C in the dark for 24 h before expression analysis.

*Renilla Luciferase Activity Assays*: All Rluc activity assays in transformed plant tissues were performed according to the manufacturer's protocol (Promega, Madison, WI, USA). *A. thaliana* leaves treated with different components in solution were collected and rinsed with water to remove the soil residues. Total leaf protein was extracted from one infiltrated leaf with 100 µL of 1 × Rluc assay lysis buffer. Fifty microliters of total protein solution was used in the Rluc activity assay reaction. For the time‐course Rluc activity assays, samples were collected at different time points and snap‐frozen in liquid N_2_ before they were stored at −80 °C for protein extraction. Approximately 2 × 2 cm^2^ leaflets of *N. benthamiana* were excised in the infiltrated area. Total protein was extracted from the leaflet with 200 µL of 1 × *Renilla* luciferase assay lysis buffer. Twenty microliters of total protein was added to 100 µL of *Renilla* luciferase assay reagent. Fruit, root, and potato tuber proteins were extracted using 100 µL of 1 × *Renilla* luciferase assay lysis buffer from ≈100 mg of powdered plant sample prepared by grinding them in liquid N_2_. Fifty microliters of protein solution was assayed with 100 µL of *Renilla* luciferase assay reagent. The measurements of relative light units (RLUs) in the Rluc assay were performed using a GloMax 20/20 luminometer (Promega). The total amount of protein in each sample was determined using a BCA Protein Assay Kit (Pierce Biotechnology, Rockford, IL, USA). The Rluc activities in samples were reported as RLU per milligram of total protein (RLU mg^−1^ protein).

*CLSM Observation and Image Analysis*: The expression of fluorescent protein in the chloroplasts of transformed *A. thaliana* and *N. benthamiana* leaves was observed using a Zeiss LSM880 confocal microscope (Carl Zeiss, Oberkochen, Germany) with an air‐objective lens (20× ) or oil emulsion‐objective lens (63× ). Sections of the leaf (0.5 × 0.5 cm^2^) infiltrated with the complex solution were cut around the syringe‐attached area. The leaf sections were washed twice with water and deaerated in water prior to CLSM observation of GFP fluorescence. The detection of GFP fluorescence was carried out at excitation/emission (Em/Ex) wavelengths of 488/500−535 nm, while the emission wavelength of 640−680 nm was used for the detection of chlorophyll autofluorescence in plant cells.

For the detection of DsRed expression in nongreen plastids, CLSM observation was performed with excitation/emission wavelengths of 561/590–600 nm. Tomato root sections were washed twice in water to remove the agar residues. One‐centimeter‐length root sections were cut off and observed for DsRed expression. Ripened‐red tomato fruit pericarps were cut into 0.5 × 0.5 cm^2^ sections with an ≈0.5 mm thickness using a razor blade. DsRed expression in the tomato fruit chromoplast was observed only in mesocarpic cells of the fruit flesh. Carotenoid autofluorescence in the chromoplasts of tomato fruits was observed with excitation/emission wavelengths of 488/500–550 nm. For potato tuber slices, the tissues were exposed to light at 25 °C with 80 µmol photons m^−2^ s^−1^ in the growth chamber after transformation to induce chlorophyll formation[qv: 4c] before washing twice with water. The tissue was sliced to a thin layer and observed for colocalization of the DsRed signal (Ex/Em: 561/590–600 nm) with chlorophyll autofluorescence in light‐induced chlorophyll‐producing amyloplasts (Ex/Em: 488/640–680 nm).[qv: 4c] The fluorescent signals of GFP and DsRed in the transfected cells were quantified using Fiji ImageJ.^35^ Fluorescent intensities were determined from at least five different regions of interest in two independent experiments. These fluorescent intensities in plastid were subtracted by the signal from background in the adjacent nontransfected cells and reported as fluorescent intensity per area (a.u./µm^2^).

*Immunoprecipitation and Western Blotting*: Chloroplast protein isolation followed by immunoprecipitation and western blotting were performed to confirm the expression of GFP in plastids. Chloroplasts were isolated from *A. thaliana* and *N. benthamiana* leaves transformed with complex solution after 24 h of incubation using a 40% Percoll gradient.^36^ Chloroplast pellets were resuspended in 1 × phosphate buffer saline (1 × PBS, pH 7.0) plus 1.0% Triton‐X100, snap‐frozen in liquid N_2_, and then thawed at 37 °C for 15 min. The chloroplast proteins were extracted from the pellet by centrifugation at 15 000 rpm for 30 min at 4 °C. Twenty‐five microliters of SureBeads protein G‐coupled magnetic beads (Bio‐Rad Laboratory, Hercules, CA, USA) was prewashed three times with 1 × PBS, pH 7.0 + 0.05% Tween‐20 (washing solution). After prewashing, 1.0 µg of rabbit anti‐GFP polyclonal antibody (NB600‐308) (Novus Biologicals, Littleton, CO, USA) was added to 400 µL of washing solution containing the magnetic beads. The antibody‐bead solution was incubated at 4 °C for 4 h with rotary shaking to facilitate the interaction between the antibody and protein G. Magnetic beads were washed three times and then resuspended in 200 µL of washing solution. Five hundred micrograms of chloroplast protein was diluted in 300 µL of washing solution and added to the magnetic bead solution. The solution was incubated at 4 °C for 16 h to facilitate the affinity interaction of GFP molecules with anti‐GFP antibody captured by protein G on the magnetic beads. After incubation, the magnetic beads were washed five times with the wash buffer, and 50 µL of 1 × Laemmli buffer supplemented with 200 × 10^−3^ m dithiothreitol (DTT) was added to the magnetic beads. The solution was denatured at 100 °C for 5 min and then magnetized before collecting the denatured protein solution in a new tube.

For western blotting, 25 µL of protein solution was resolved in 8%−16% Mini‐PROTEAN Precast protein gels (Bio‐Rad Laboratory) at 80 V for 2 h. Protein was blotted onto a polyvinylidene difluoride (PVDF) membrane with a Trans‐Blot SD Semi‐Dry transfer cell (Bio‐Rad Laboratory) at 10 V for 1 h. The membrane was incubated with a blocking solution containing 5% skim milk in 1 × PBS, pH 7.0 + 0.05% Tween‐20 at room temperature for 1 h. After blocking, the membrane was transferred into a primary antibody solution containing 1:1000 rabbit anti‐GFP polyclonal antibody (NB600‐308) (Novus Biologicals) and then into a secondary antibody solution containing 1:20 000 horse radish peroxidase (HRP) conjugated goat anti‐rabbit IgG polyclonal antibody (ab6721) (Abcam, Tokyo, Japan). After incubation, the membrane was washed five times with 1 × PBS, pH 7.0 + 0.05% Tween‐20. The signal of HRP activity on the membrane was detected by adding 1 mL of SuperSignal West Pico PLUS chemiluminescent substrate (Thermo Scientific, Waltham, MA, USA) onto the membrane and then visualized using a LAS3000 imaging system (Fujifilm, Tokyo, Japan).

*Internalization and Plastid‐Targeted Delivery of pDNA Molecules Mediated by CTP/CTP Complexes*: Plasmid DNA p*PpsbA::Rluc* was labeled with Cy3 fluorescent dye using a *Label* IT Nucleic acid Labeling Kit (Mirus Bio, Madison, WI, USA) according to the manufacturer's protocol. A solution of Cy3‐labeled pDNA/CTP/CPP complexes was introduced into the fully expanded leaves of *N. benthamiana* (6 weeks old) using a needleless syringe. The localization of Cy3‐labeled pDNA in the chloroplast and the colocalization of Cy3‐labeled pDNA/CTP/CPP complexes with FM4‐64 were observed after 2 h of incubation by CLSM imaging with a 555/560–580 nm (Ex/Em) wavelength for Cy3 detection, a 488/640–680 nm (Ex/Em) wavelength for the chlorophyll autofluorescence, and a 514/680–700 nm (Ex/Em) wavelength for FM4‐64 detection.

*Particle Bombardment*: Thirty‐five milligrams of 0.6 µm gold particles (Bio‐Rad Laboratory) was coated with 10 µg of p*PpsbA*::*Rluc* or p*Prrn*::*GFP(S65T)*::*TpsbA* with 50 µL of 2.5 m CaCl_2_ and 20 µL of 0.1 m spermidine. Four‐week‐old *Arabidopsis* leaves (20–25 leaves) grown on soil were washed five times with water and then placed abaxial side‐up on ½ MS agar supplemented with 1.0% sucrose. Tomato fruit slices were dissected from the fruits with razor blade to ≈0.5 mm × 1.0 cm × 1.0 cm dimensions and placed on the MS + 3.0% sucrose agar media. The DNA‐coated gold particles were bombarded to the *Arabidopsis* leaves and tomato fruit slices using Biolistic PDS‐1000/He Particle delivery system with 1100 psi rupture disc (Bio‐Rad Laboratory). Transformed plant tissues were incubated at standard culture conditions in the growth chamber for further expression analyses with Rluc assays and CLSM observation. Gold particles coated with p*P35S::DsRed::TNos* were bombarded to the *Arabidopsis* leaves as the constitutive cytoplasmic‐expression control.

*Statistical Analyses*: The significant differences of all data were compared using analysis of variance (ANOVA) and Tukey's honestly significant difference (HSD) test with a *p* value equal to 0.05 or a comparative Student's *t‐test* performed by the StatPlus:mac statistical software (AnalystSoft, Walnut, CA, USA). Unless mentioned otherwise, the data are presented as the average value of three individual experiments.

## Conflict of Interest

The authors declare no conflict of interest.

## Supporting information

Supplementary

Supplementary
