# Supplementary material for: Targeted Gene Delivery into Various Plastids Mediated by Clustered Cell‐Penetrating and Chloroplast‐Targeting Peptides
Source: Adv Sci (Weinh). 2019 Oct 23;6(23):1902064. doi: 10.1002/advs.201902064 (PMC6891901; doi:10.1002/advs.201902064)
Supplement: Supplementary file 1 — Supplementary [file ADVS-6-1902064-s001.pdf]

Copyright WILEY-VCH Verlag GmbH & Co. KGaA, 69469 Weinheim, Germany, 2019.

ADVANCED SCIENCE
Open Access

# Supporting Information

for *Adv. Sci.,* DOI: 10.1002/advs.201902064

# Targeted Gene Delivery into Various Plastids Mediated by Clustered Cell-Penetrating and Chloroplast-Targeting Peptides

*Chonprakun Thagun, Jo-Ann Chuah, and Keiji Numata**

((Supporting Information can be included here using this template))

Copyright WILEY-VCH Verlag GmbH & Co. KGaA, 69469 Weinheim, Germany, 2016.

# Supporting Information

**Title** Targeted gene delivery into various plastids mediated by clustered cell-penetrating and chloroplast-targeting peptides.

*Chonprakun Thagun, Jo-Ann Chuah, and Keiji Numata* *

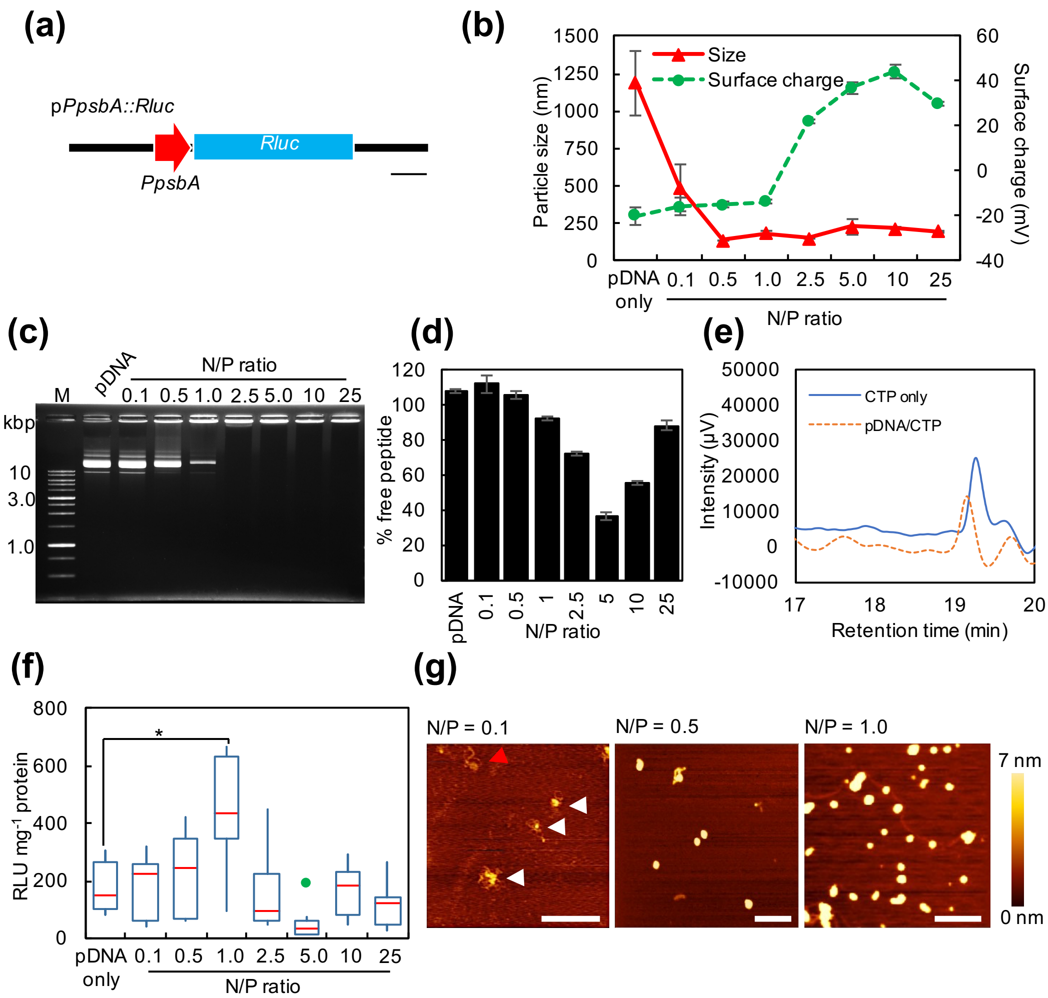


**Figure S1. Characterization of pDNA(p*PpsbA::Rluc*)/CTP (KH$_9$-OEP34) complexes.** (**a**) Expression cassette of the *Renilla luciferase* (*Rluc*) gene driven by the light-inducible *psbA* gene promoter (*PpsbA*) for specific transgene expression in the plant plastids. Bar = 200 bp. (**b**) Particle size and surface charge of the pDNA/KH$_9$-OEP34 complexes formed at

different peptide-to-plasmid DNA ratios (N/P ratio) analyzed by a Zeta Nanosizer. Error bars represent the SD of the average value from 3 replicates. (**c**) Electrophoretic mobility-shift assays of the pDNA/$KH_9$-OEP34 complexes at various N/P ratios. (**d**) Abundance of free $KH_9$-OEP34 in the pDNA/CTP complex solution at different N/P ratios. Error bars represent the standard deviation (SD) of the average intensity from three replicates. (**e**) HPLC chromatograms of free polypeptide in the solutions with and without the pDNA/CTP complex formation at N/P ratio = 1.0. (**f**) *Renilla* luciferase activity in *Arabidopsis thaliana* leaves syringe-infiltrated with the p*PpsbA::Rluc*/$KH_9$-OEP34 complexes formed at different N/P ratios at 24 hours post-infiltration. The data are presented in the form of a box plot. Red lines represent the median value of data from 8 independent biological samples ($n$ = 8). Maximum and minimum values are shown by the upper and lower bars. The green dot shows the outlier in the distribution of data. The asterisk indicates the significant difference of the mean value compared to that of the pDNA-only control treatment analyzed by Student's *t-test* at $p < 0.05$. (**g**) AFM images of the pDNA/CTP complexes at N/P ratios of 0.1, 0.5 and 1.0. Scale bars = 500 nm. The heat map represents the height of complexes on the mica surface.

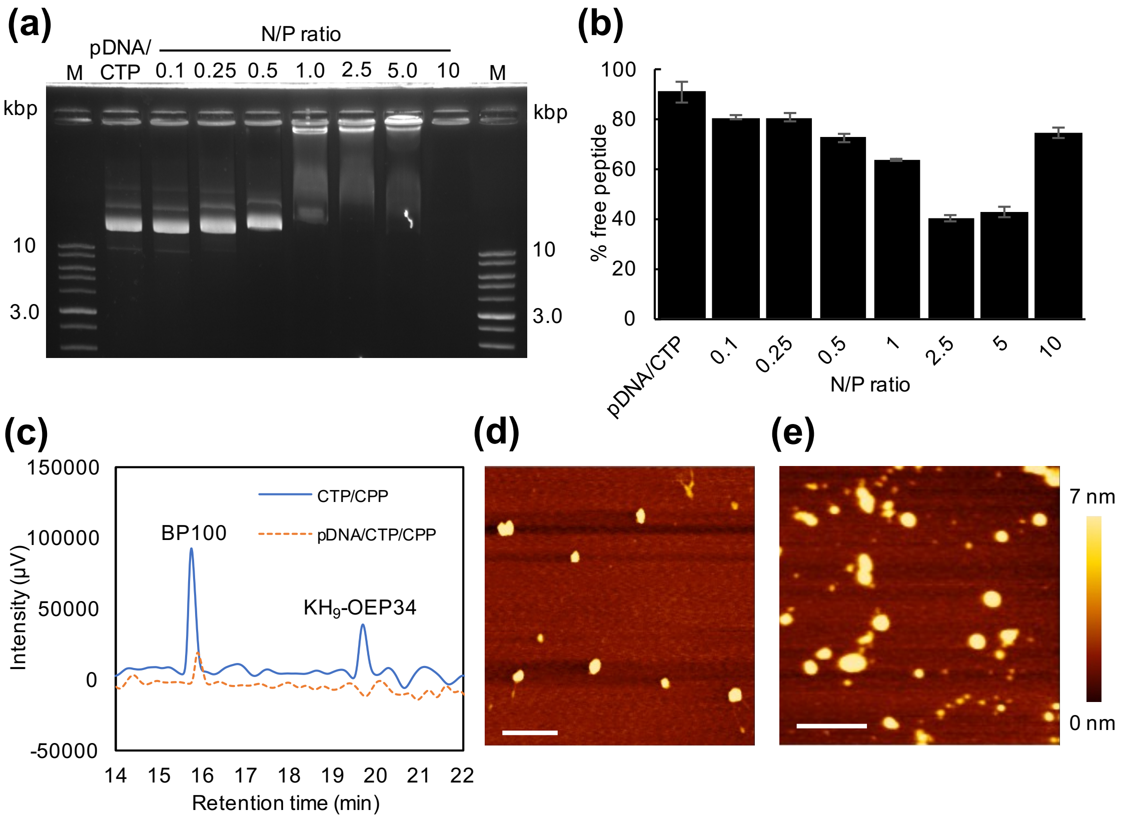


**Figure S2. Gel mobility shifts and size distribution of the clustered pDNA/$KH_9$-OEP34/BP100 complexes.**

(**a**) Agarose gel electrophoresis of the pDNA(p*PpsbA::Rluc*)/$KH_9$-OEP34/BP100 complexes formed at various N/P ratios. (**b**) Abundance of free peptides in the pDNA/CTP/CPP complex solution formed at different N/P ratios. Error bars represent the standard deviation (SD) of the average intensity from three replicates. (**c**) HPLC chromatogram of polypeptide solution with and without pDNA molecule at N/P ratio = 1.0. (**d**) and (**e**) Morphologies of the pDNA/CTP/CPP complexes formed at N/P ratios of 0.5 (**d**) and 2.5 (**e**). Scale bars = 500 nm. The heat map represents the height of complexes on the mica surface.

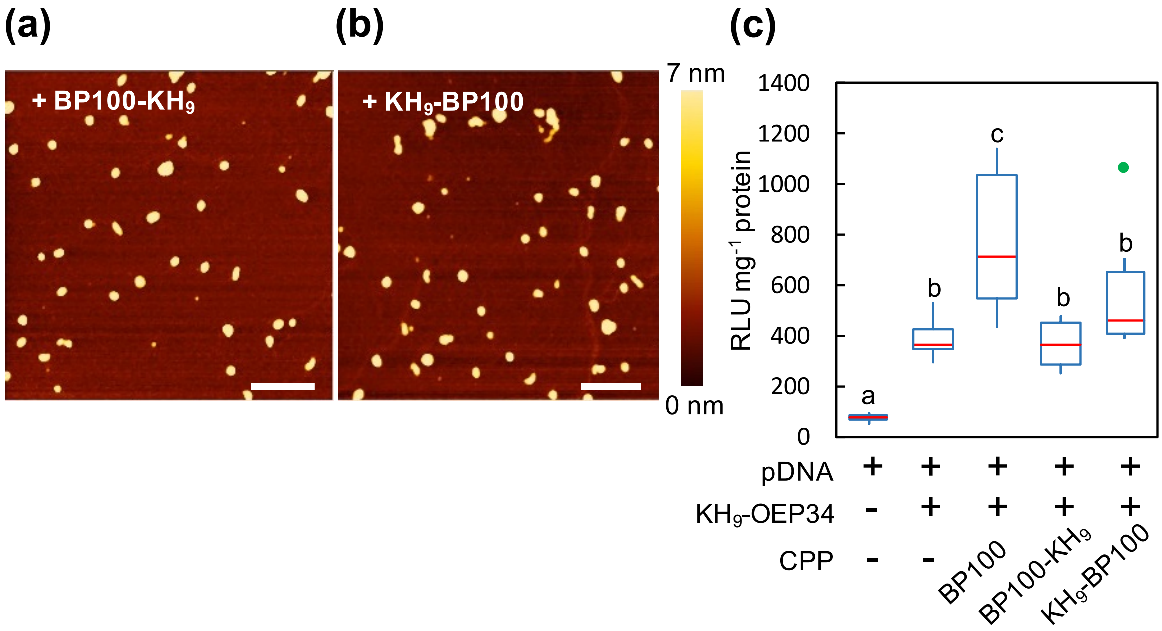


**Figure S3. Efficiencies of the clustered pDNA/CTP/CPP complexes formulated using different BP100 derivatives for plastid transformation.**
(**a**) and (**b**) Morphologies of the clustered pDNA/CTP/CPP complexes formulated using BP100 derivatives, BP100-$KH_9$ or $KH_9$-BP100, at an N/P ratio of 1.0 observed by AFM. Scale bars = 500 nm. The heat map represents the height of complexes on the mica surface. (**c**) Transformation efficiencies of the different clustered complexes formed using different BP100 derivatives (as the CPP). The distribution of Rluc activity in six *N. benthamiana* leaves ($n = 6$) transformed with a complex solution or a solution containing pDNA alone as a control at 24 hours post-infiltration is shown in the form of a box plot. Red lines indicate the median value. The green dot shows the outlier in the distribution of data. The letters indicate the significant difference of the average Rluc activity among samples infiltrated with different complex solutions analyzed by one-way ANOVA and Tukey's HSD test at $p = 0.05$.

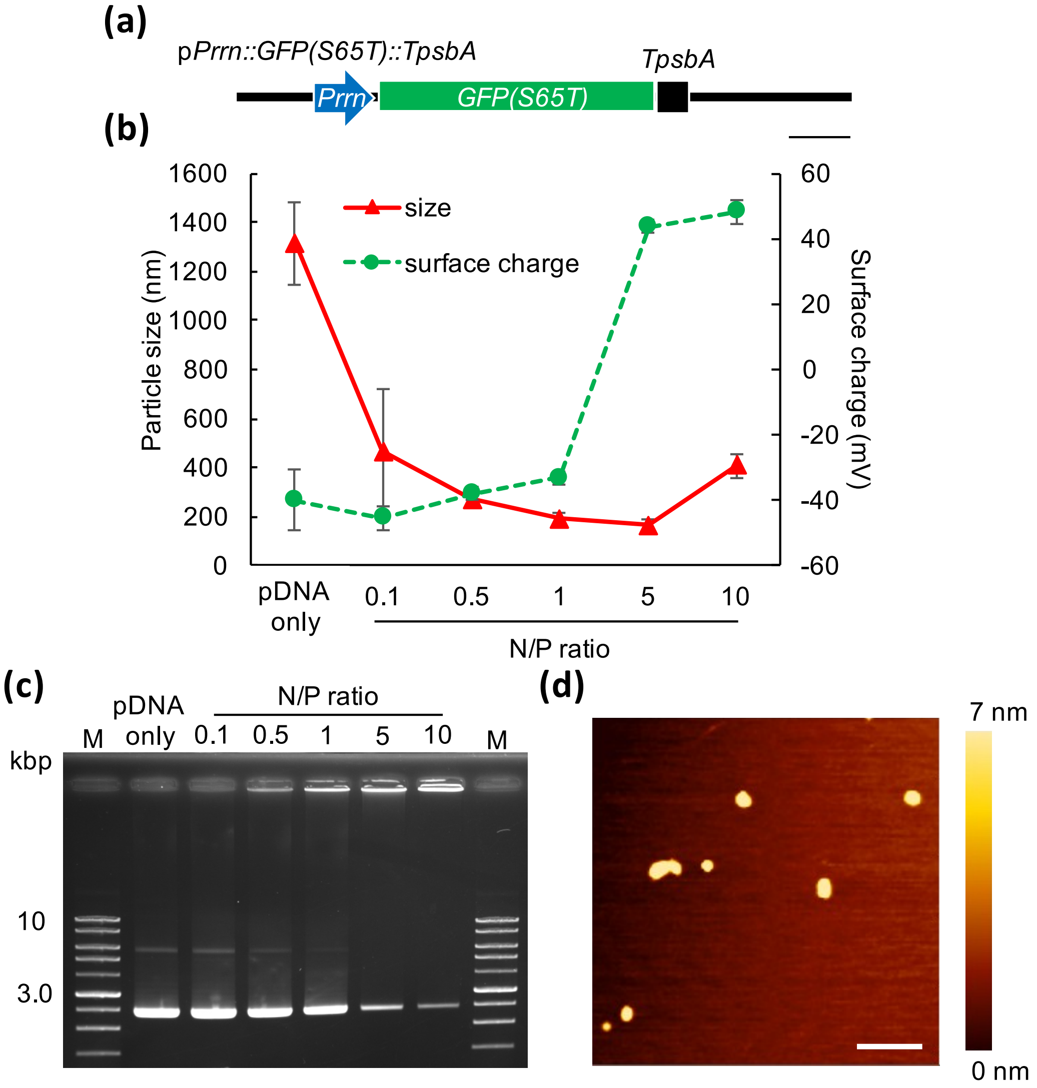


**Figure S4. Characterization of the pDNA(p*Prrn::GFP(S65T)::TpsbA*)/CTP (KH$_9$-OEP34) complexes for fluorescent reporter protein expression in plastids.**
(**a**) Expression cassette for GFP expression in plastids. The *GFP(S65T)* gene was transcriptionally fused to the plastid-specific *rrn* promoter and *psbA3'-UTR* sequence (*TpsbA*). Bar = 200 bp. (**b**) Particle size and surface charge of the pDNA/KH$_9$-OEP34 complexes formed at various N/P ratios. Error bars show the SD of the average value from 3 replicates. (**c**) Electrophoretic mobility-shift assay of the pDNA(p*Prrn::GFP(S65T)::TpsbA*)/KH$_9$-OEP34 complexes at different N/P ratios. (**d**) AFM imaging of pDNA/KH$_9$-OEP34 complexes formed at an N/P ratio of 1.0. Scale bar = 500 nm. The heat map represents the height of complexes on the mica surface.

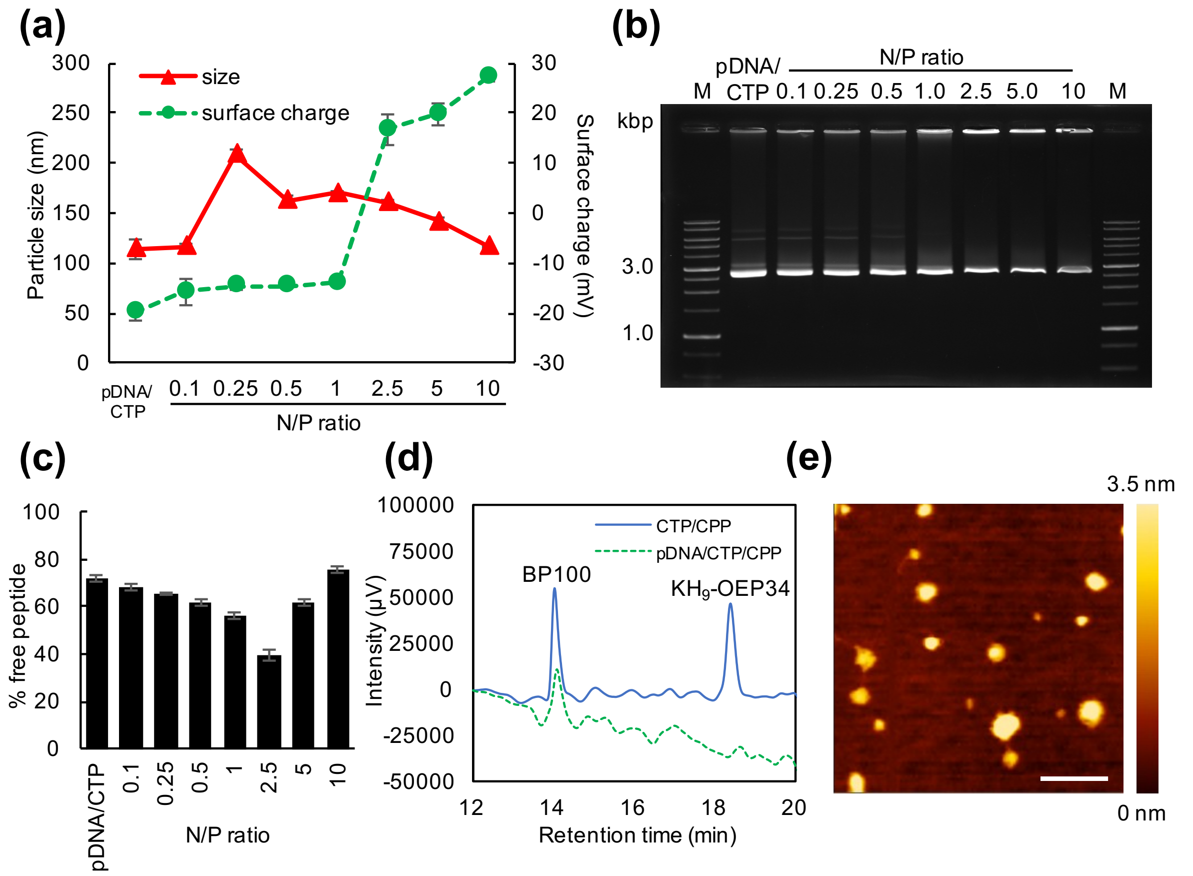


**Figure S5. Characterization of the clustered pDNA(p*Prrn::GFP(S65T)::TpsbA*)/KH$_9$-OEP34/BP100 complexes for fluorescent reporter protein expression in plastids.**
(**a**) Particle size and surface charge of pDNA/KH$_9$-OEP34/BP100 complexes formed at different N/P ratios. Error bars represent the SD of the average value from three replicates. (**b**) Electrophoretic mobility-shift assay of pDNA/KH$_9$-OEP34/BP100 complexes formed at different N/P ratios. (**c**) Abundance of free peptide in the pDNA/CTP/CPP complex solution formed at different N/P ratios. Error bars represent the standard deviation (SD) of the average intensity from three replicates. (**d**) HPLC chromatogram of polypeptide solution with and without pDNA molecule at N/P ratio = 1.0. (**e**) AFM imaging of the pDNA/CTP/CPP complexes formed at an N/P ratio of 1.0. Scale bar = 500 nm. The heat map shows the height of complexes on the mica surface.

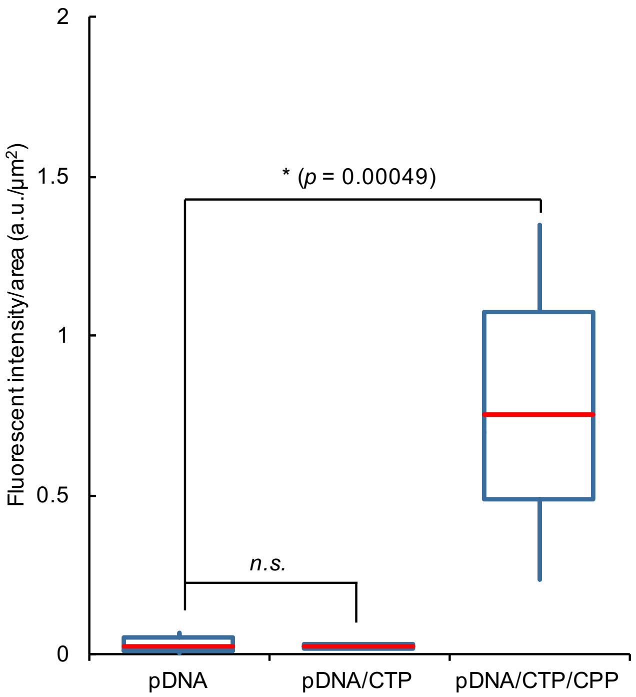


**Figure S6. GFP fluorescence in *Arabidopsis* leaf cells transfected by pDNA/peptide complexes.**

The expression of GFP was observed by CLSM at 24 hours post-transfection. The CLSM images were taken from 5 regions of interest (ROIs) of 5 pDNA only-treated and pDNA/CTP complex-infiltrated leaves ($n$ = 5), and 10 ROIs of 5 pDNA/CTP/CPP complex-infiltrated leaves ($n$ = 10). The fluorescent intensities in these images were determined and normalized per area of observation (a.u./$\mu m^2$). The distributions of fluorescent intensity per area were represented by box plot. Red lines represent mean of data. The asterisk indicates the significant difference of mean analyzed by Student's *t-test*. n.s. shows non-statistically difference between two treatments at $p < 0.05$.

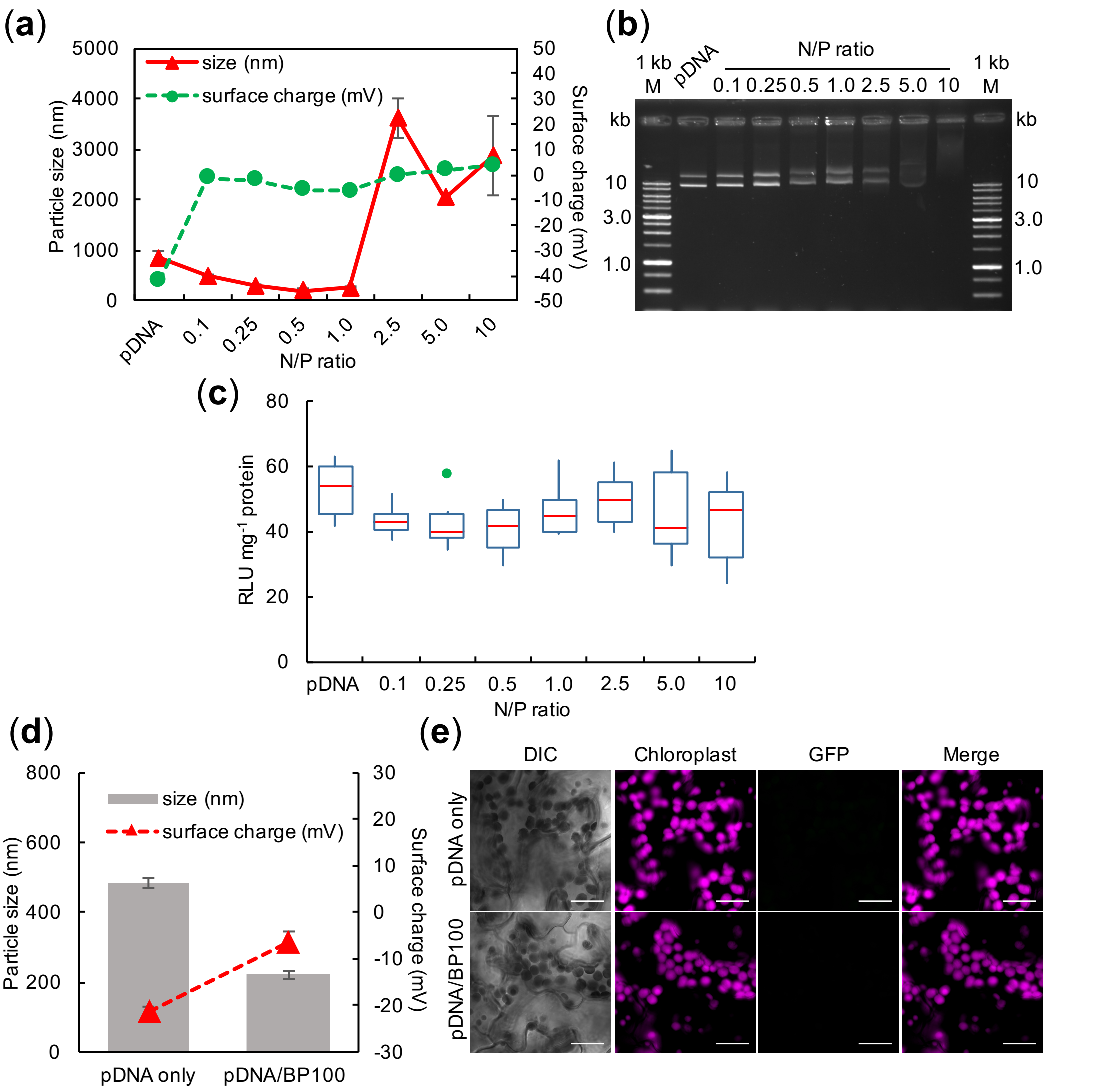


**Figure S7. Performance of pDNA/BP100 complex in plastid-targeting gene delivery.** (a) Particle size and surface charge of pDNA/BP100 complexes formed at various N/P ratios. Error bars represent the standard deviations of the average value from 3 replicates. (b) Electrophoretic mobility shifts of p*PpsbA::Rluc*/BP100 complexes at different N/P ratios. (c) Luciferase activities in p*PpsbA::Rluc*/BP100 complex-transfected Arabidopsis leaves. The distribution of Rluc activities in 8 Arabidopsis leaves transfected with the pDNA/BP100 complex at 24 hours post-transfection is shown as the form of box plot. Red lines represent the median values. The green dot indicates the outlier in the distribution of data. (d) Particle size and surface charge of p*Prrn::GFP(S65T)::TpsbA*/BP100 complex formed at N/P ratio = 1.0. Error bars represent the standard deviation (SD) of the average value from three replicates. (e) CLSM observation of GFP in *Arabidopsis* leaves at 24 hours post-infiltration with p*Prrn::GFP(S65T)::TpsbA*/BP100 complexes. Scale bars = 20 µm.

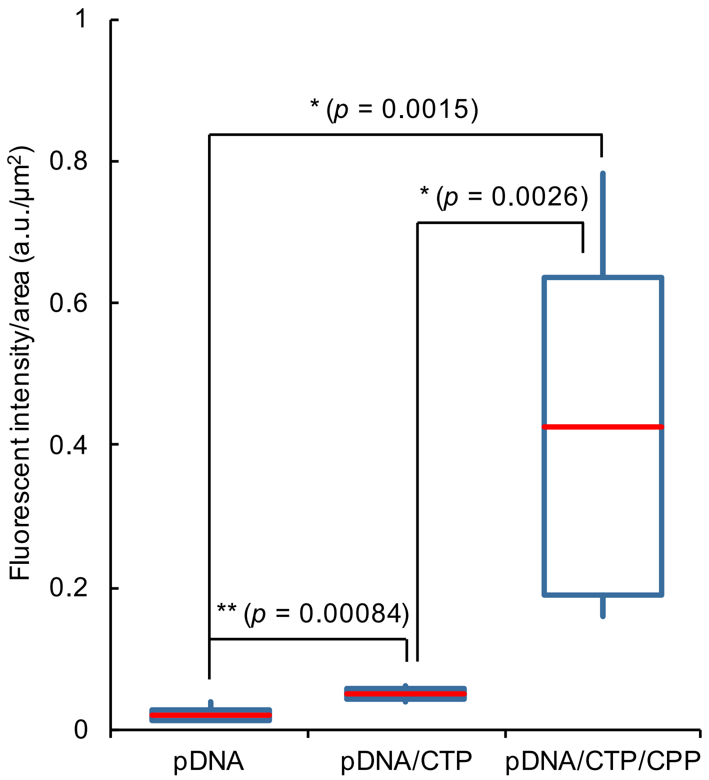


**Figure S8. GFP expression in *Nicotiana benthamiana* leaf cells transfected by pDNA/CTP/CPP complexes.**

The expression of GFP was observed by CLSM at 24 hours post-transfection. The CLSM images were taken from 5 regions of interest (ROIs) of 5 pDNA-only-treated and pDNA/CTP complexes-infiltrated leaves ($n$ = 5), and 10 ROIs of 5 pDNA/CTP/CPP complexes-infiltrated leaves ($n$ = 10). The fluorescent intensities in these images were determined and normalized per area of observation (a.u./$\mu m^2$). The distributions of fluorescent intensity per area were represented by box plot. The red lines represent mean of data. The asterisks indicate the levels of statistical difference of mean analyzed by Student's *t-test*. * = $p < 0.01$, ** = $p < 0.001$.

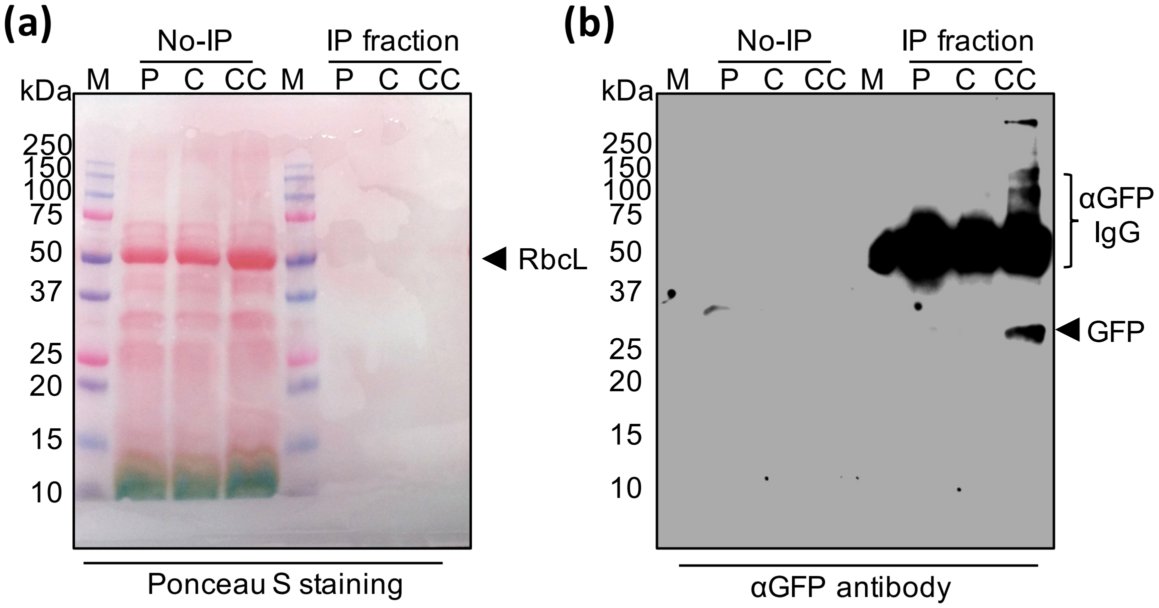


**Figure S9. Immunoprecipitation and western blotting for the detection of GFP in the chloroplasts of *N. benthamiana* delivered using the clustered pDNA/CTP/CPP complexes.** (**a**) Ponceau S-stained membrane after protein transfer onto the PVDF membrane determined by equal loading of protein isolated from the chloroplasts of complex-transformed plant leaves (No-IP) and immunoprecipitated proteins against anti-GFP antibody (IP fraction). The arrow indicates the equal amount of the large subunit of the Rubisco protein (RbcL) as an internal control. (**b**) Complete immunoblot image for **Figure 4b**. In (**a**) and (**b**), M is the dual-colored Precision Plus® protein marker (Bio-Rad Laboratory). P is the protein sample isolated from the chloroplasts of plants infiltrated with pDNA only, C represents the pDNA/CTP complex, and CC stands for the clustered pDNA/CTP/CPP complex-transformed plant tissues. The arrow indicates the GFP band in the image after exposure to LAS3000 for 15 min.

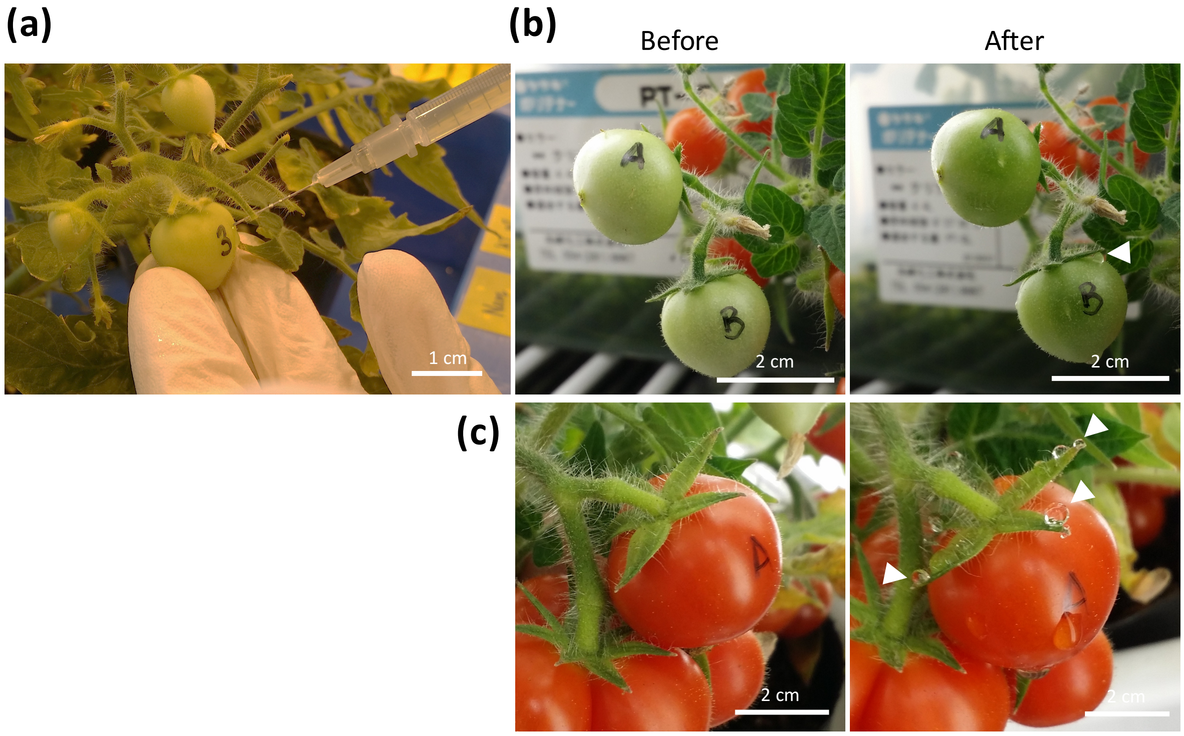


**Figure S10. Tomato fruit injection.**

(**a**) Introduction of the complex solution into the mature-green tomato fruit by injection. Mature-green fruits (~ 1.5 – 2 cm in diameter) of *Solanum lycopersicum* (*cuv*. MicroTOM) grown in the plant growth chamber were injected with a solution containing the clustered pDNA/CTP/CPP complexes using a needle-attached syringe. A similar protocol was used for the ripened-red fruits. (**b**) and (**c**) Complete introduction of the complex solution into the tomato fruit was determined by excess amounts of solution at the sepal tips of the injected fruits (indicated by arrows) after injection of a mature-green fruit (**b**) and a ripened-red fruit (**c**)). The fruit color was also different after the injection process, indicating complete dispersion of the solution in the transformed fruit.

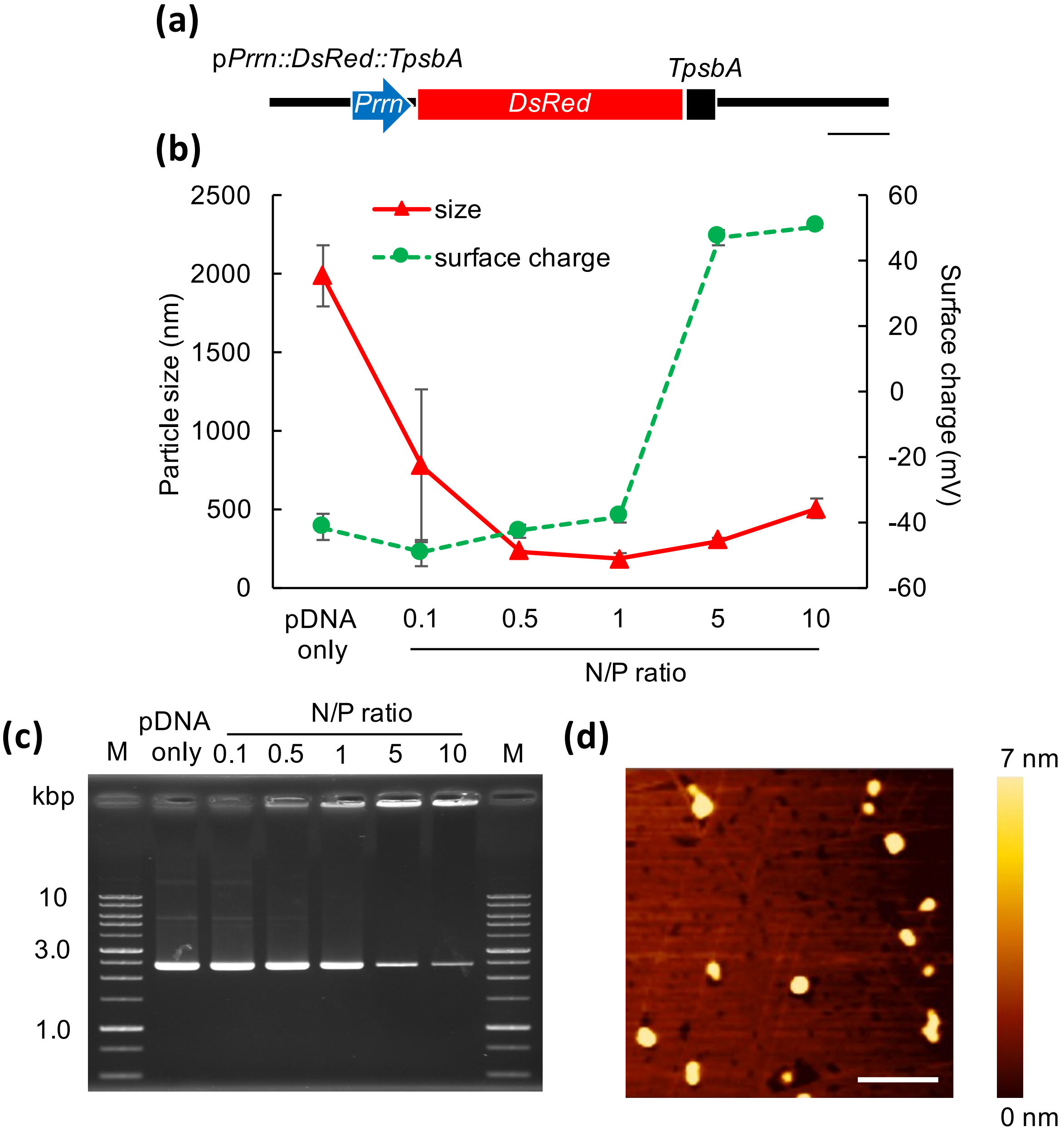


**Figure S11. Characterization of the pDNA(p*Prrn::DsRed::TpsbA*)/$KH_9$-OEP34 complexes.**

(**a**) Expression cassette for DsRed expression in plastids. Bar = 200 bp. (**b**) Particle size and surface charge of the pDNA/$KH_9$-OEP34 complexes formed at various N/P ratios. Error bars show the SD of the average value from 3 replicates. (**c**) Electrophoretic mobility-shift assay of the pDNA/$KH_9$-OEP34 complexes at different N/P ratios. (**d**) AFM imaging of the pDNA/$KH_9$-OEP34 complexes formed at an N/P ratio of 1.0. Scale bar = 500 nm. The heat map represents the height of complexes on the mica surface.

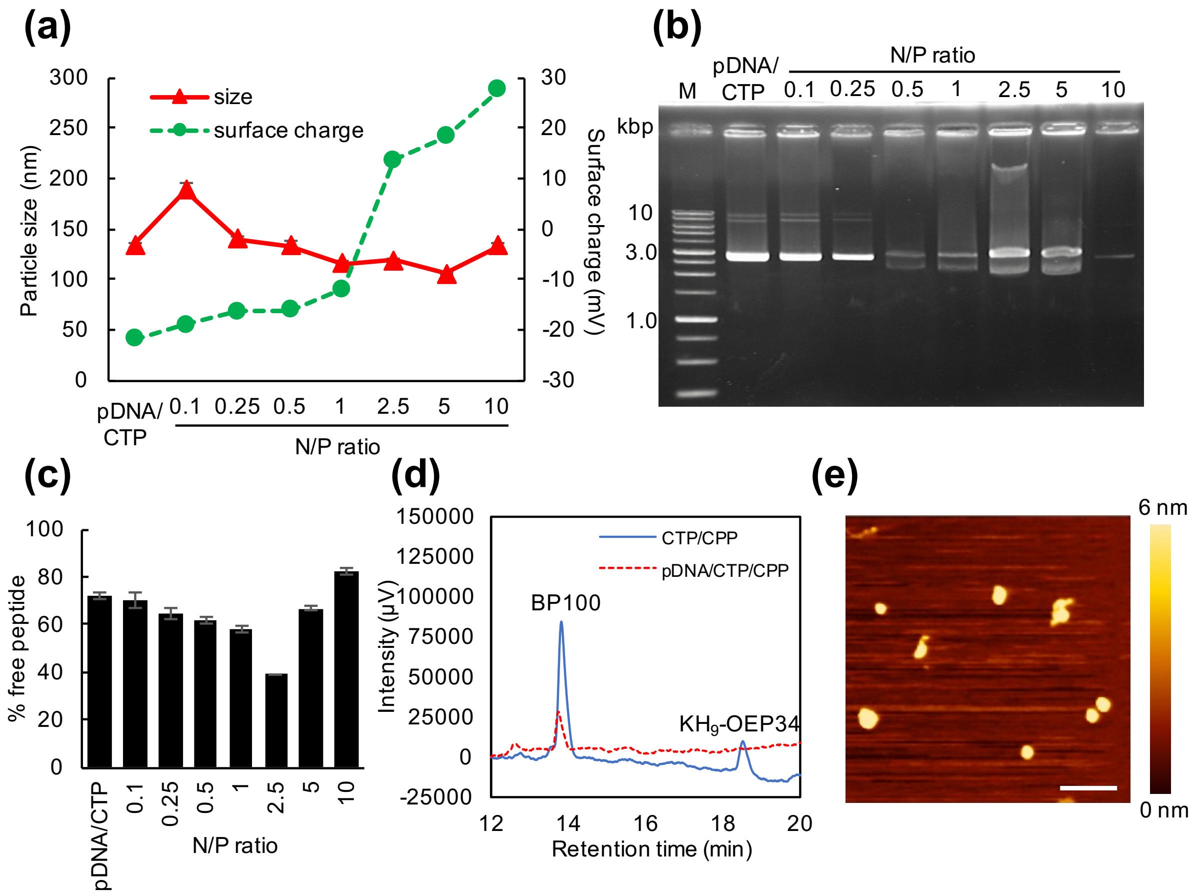


**Figure S12. Physiological properties of the clustered pDNA(p*Prrn::DsRed::TpsbA*)/KH$_9$-OEP34/BP100 complexes for fluorescent reporter expression in plastids.**

Particle size and surface charge of the pDNA/KH$_9$-OEP34/BP100 complexes formed at various N/P ratios. Error bars represent the SD of the average value from three replicates. (**b**) Gel-retardation assay of the pDNA/KH$_9$-OEP34/BP100 complexes at different N/P ratios. (**c**) Abundance of free peptide in the pDNA/CTP/CPP complex solution formed at different N/P ratios. Error bars represent the standard deviation (SD) of the average intensity from three replicates. (**d**) HPLC chromatogram of polypeptide solution with and without pDNA at N/P ratio = 1.0. (**e**) AFM imaging of the pDNA/KH$_9$-OEP34/BP100 complexes formed at an N/P ratio of 1.0. Scale bar = 500 nm. The heat map shows the height of complexes on the mica surface.

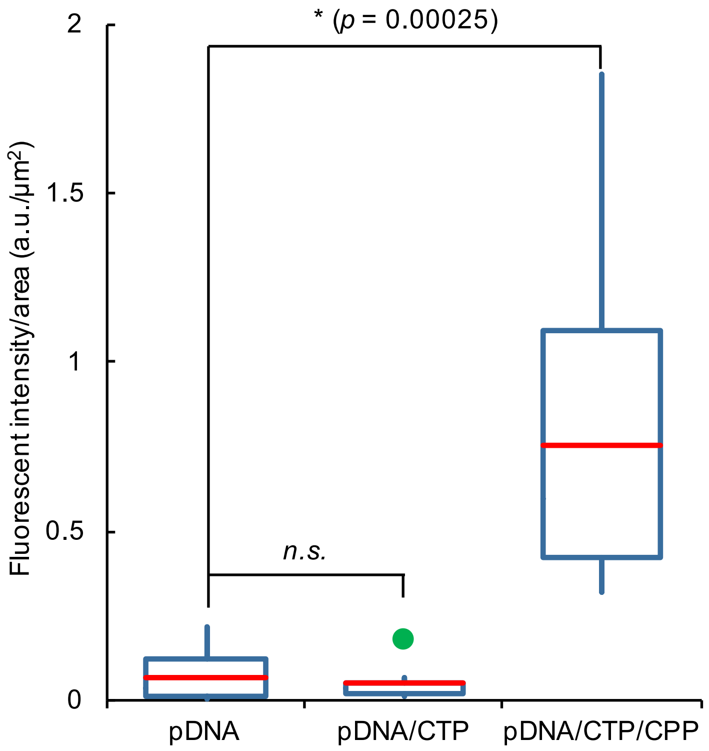


**Figure S13. DsRed overexpression in tomato pericarpic cells transfected by pDNA/CTP/CPP complexes.**

DsRed expression in tomato pericarpic cells were observed by the CLSM at 24 hours post-infiltration with pDNA/peptide complexes. The distributions of red fluorescent intensity per area in 5 ROIs taken from pDNA only-treated and pDNA/CTP complex-infiltrated fruits ($n$ = 5) and 10 ROIs from pDNA/CTP/CPP complex-injected fruits ($n$ = 10) were represented by box plot. The red lines indicate the mean of data. The green dot shows the outlier of the distribution of data. The asterisk represents the significant difference of means between the treatments analyzed by Student's *t-test*. n.s. = not significantly different at $p < 0.05$.

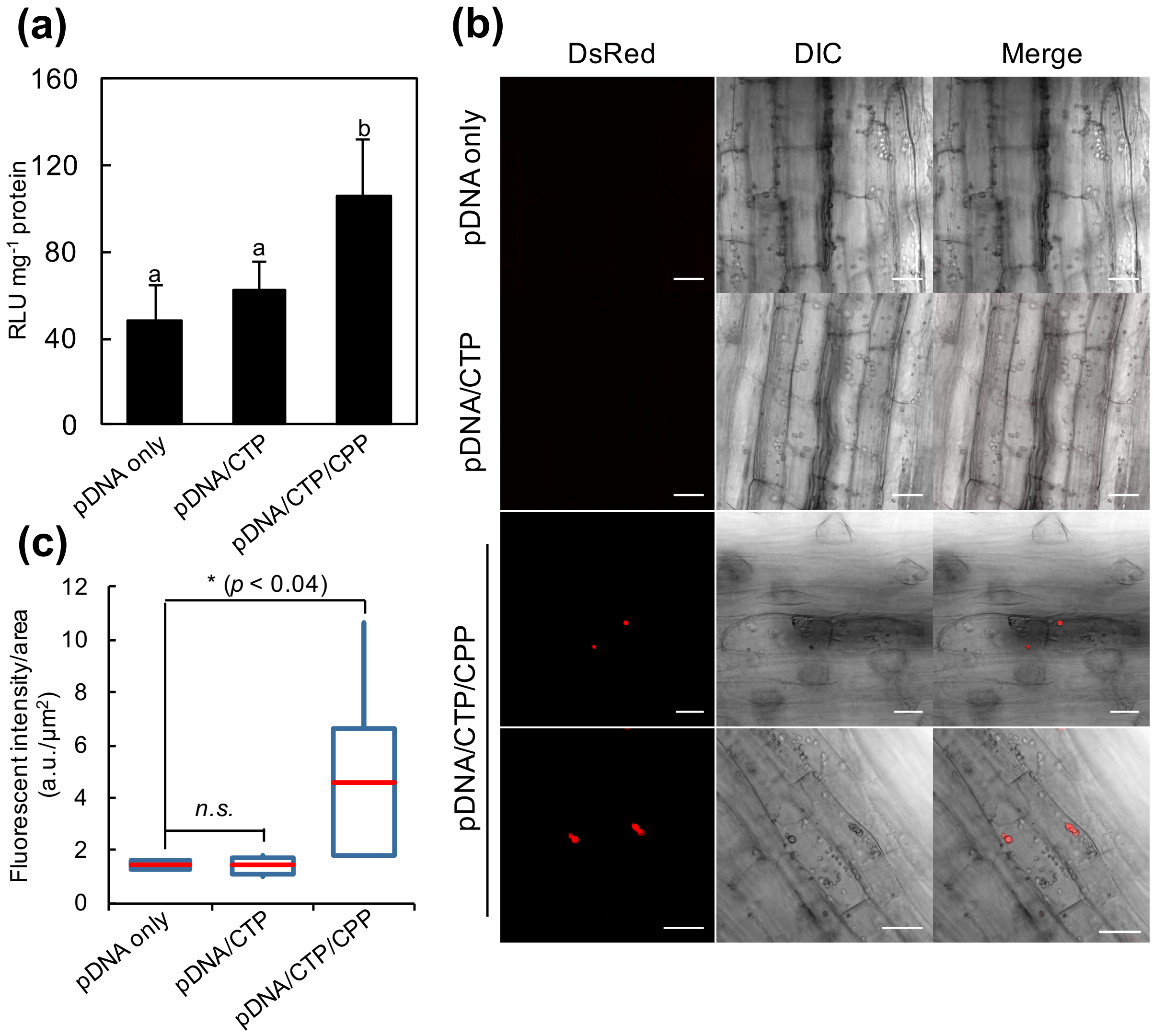


**Figure S14. Plastid transformation mediated by the clustered CTP/CPP complexes in tomato roots.**

(**a**) *Renilla* luciferase activities in tomato roots transformed with p*PpsbA::Rluc* only, pDNA/CTP complexes and clustered pDNA/CTP/CPP complexes at 24 hours post-infiltration. Error bars represent the SD of the average Rluc activities in 4 biological independent samples ($n = 4$) for each treatment. Letters indicate the significant difference of means among the treatment groups analyzed by one-way ANOVA and Tukey's HSD test at $p = 0.05$. (**b**) CLSM imaging of plastids overexpressing DsRed in tomato root cells. A clustered pDNA(p*Prrn::DsRed::TpsbA*)/$KH_9$-OEP34/BP100 complex solution and solutions containing pDNA only or pDNA/CTP complexes were introduced into the root segments of 7-day-old tomato seedlings by vacuum infiltration. CLSM observation was performed 24 hours after infiltration. Scale bars = 20 μm. (**c**) Quantitative DsRed expression analysis of root cells

transfected with pDNA/peptide complexes. The expression of DsRed was observed by CLSM at 24 hours post-infiltration. The box plot represents the distributions of fluorescent intensity per area in 5 pDNA only- and pDNA/CTP complex-transfected roots ($n = 5$), and 10 pDNA/CTP/CPP complex-transfected roots ($n = 10$). Red lines indicate the mean of data. The asterisk represents the significant difference of means analyzed by Student's *t-test*. n.s. = not significant different at $p < 0.05$.

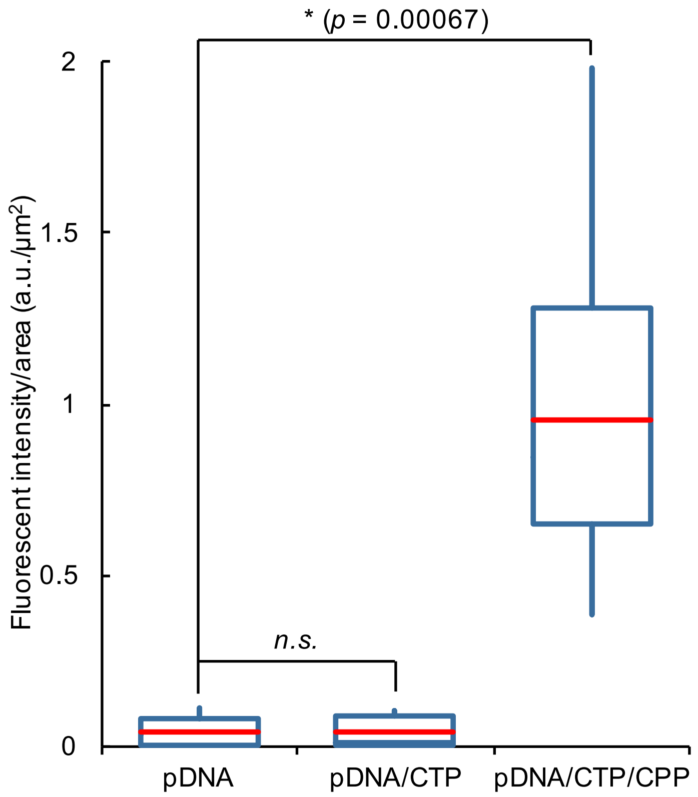


**Figure S15. DsRed expression in the amyloplasts in potato tuber cells.**

DsRed expression in potato tuber cells were observed by the CLSM at 24 hours post-infiltration with pDNA/peptide complexes. The distributions of the red fluorescent intensities in 5 ROIs taken from pDNA only-treated and pDNA/CTP complex-infiltrated fruits ($n = 5$) and 10 ROIs from pDNA/CTP/CPP complex-injected fruits ($n = 10$) were represented by box plot. Red line indicates the mean of data. The asterisk represents the significant difference of means between treatments analyzed by Student's *t-test*. n.s. = not significantly different at $p < 0.05$.

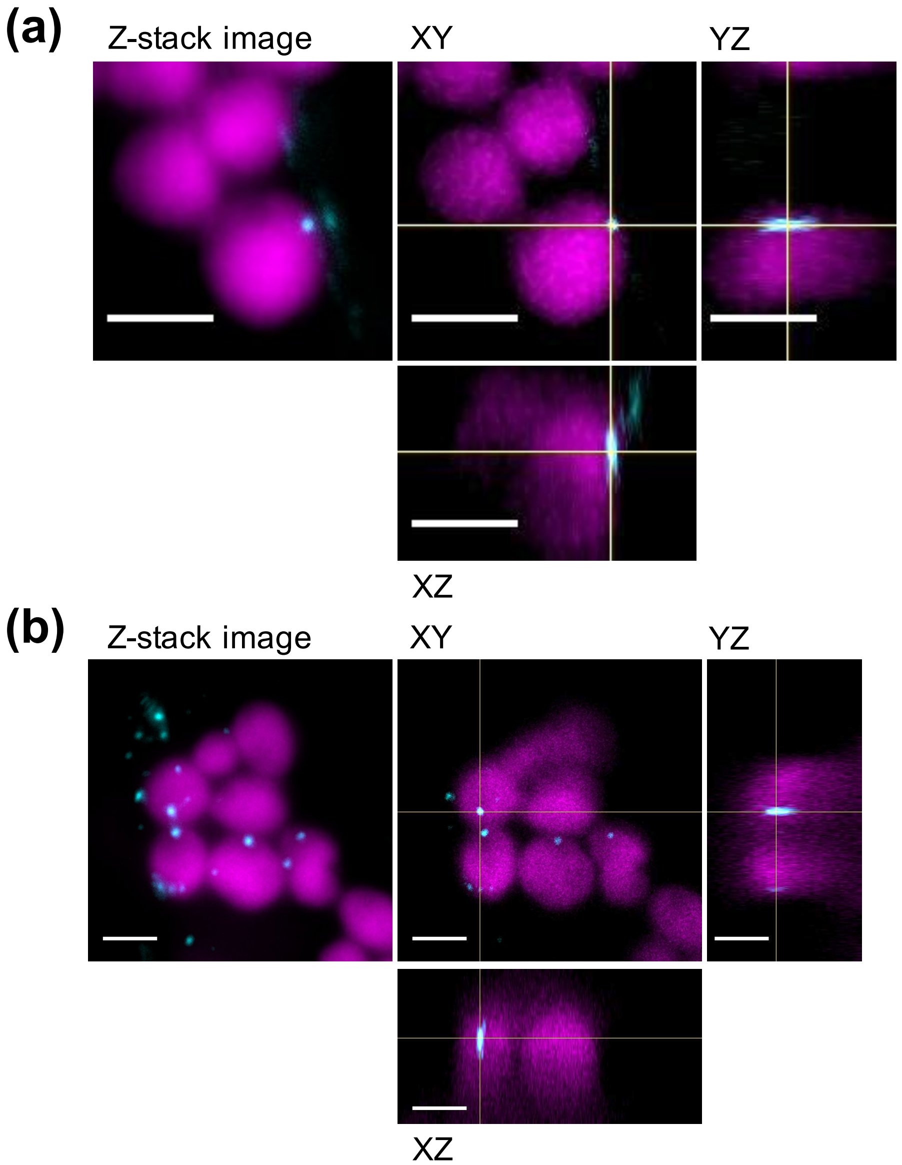


**Figure S16. Localization of Cy3-labeled pDNA molecules inside chloroplasts.** Z-stack imaging of Cy3 fluorescence colocalized with the chloroplasts of an *N. benthamiana* leaf transformed with clustered Cy3-labeled pDNA/CTP/CPP complexes at 2 hours post-infiltration (**a**) Association of the clustered Cy3-labeled pDNA/CTP complexes with the chloroplast membrane. (**b**) Observation of the clustered Cy3-labeled pDNA molecule inside the chloroplasts of leaf cells transformed with the clustered Cy3-pDNA/CTP/CPP complexes. Scale bars = 5 µm.

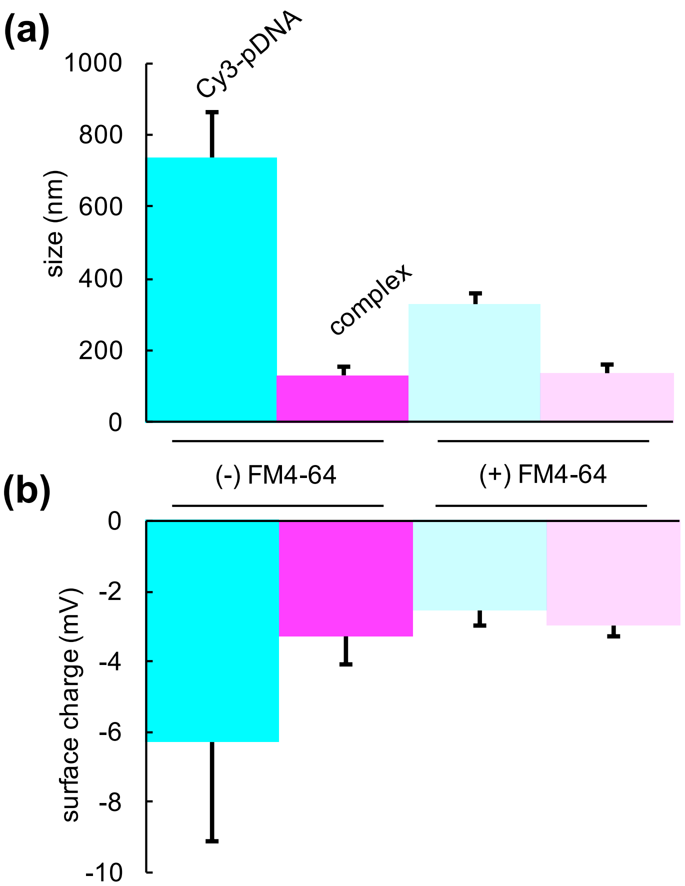


**Figure S17. Effects of FM4-64 on the size and surface charge of plasmid DNA and clustered pDNA/CTP/CPP complexes.**

Particle size (**a**) and surface charge (**b**) of the Cy3-labeled p*PpsbA::Rluc* (pDNA)/CTP/CPP complexes (complex) and free Cy3-pDNA molecule (Cy3-pDNA) in a solution with (+) or without (-) 10 µm FM4-64 analyzed by a Zeta Nanosizer. Error bars represent the SD of the average value from three replicates.

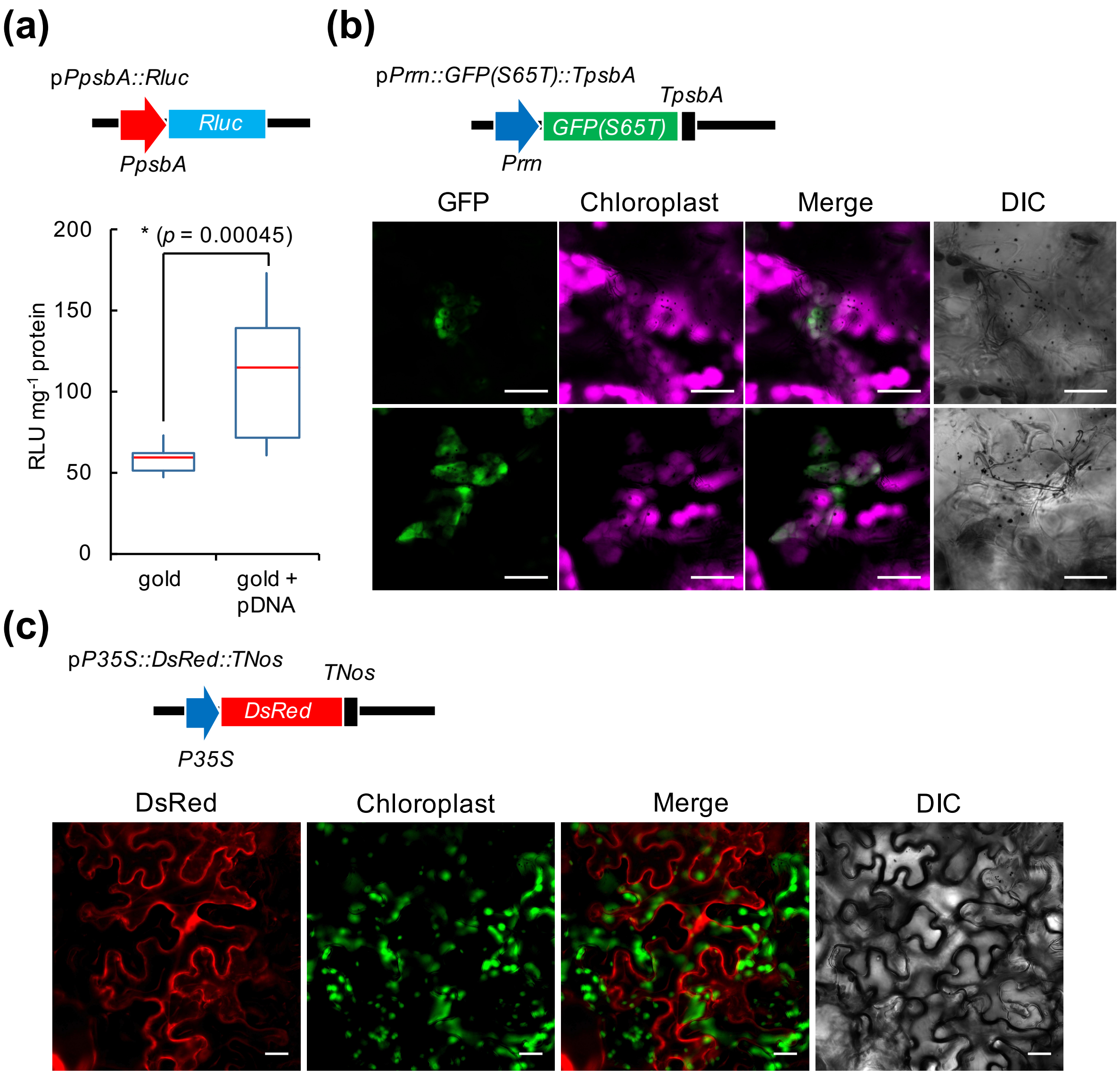


**Figure S18. Expressions of reporter proteins in plant cells mediated by biolistic particle bombardment.**

(**a**) *Renilla* luciferase activity in Arabidopsis leaves transfected with gold particle only (gold) or gold particle coated with p*PpsbA::Rluc* (gold + pDNA) at 24 hours post-transfection. The distribution of luciferase activities in plant leaves was shown as the box plot. Red lines represent the median of data distribution. The asterisk indicates the statistical significant difference between two treatments analyzed by Student's *t-test* ($n$ = 10). (**b**) Expression of GFP in the chloroplasts of Arabidopsis leaf cells mediated by particle bombardment. Arabidopsis leaves were bombarded with gold particles coated with p*Prrn::GFP(S65T)::TpsbA*. The CLSM observation were carried out at 24 hours post-transfection. (**c**) Expression of DsRed in cytoplasm of plant cells at 24 hours post-transfection with p*P35S::DsRed::TNos*-coated gold particles using particle bombardment. Scale bars = 20 µm.

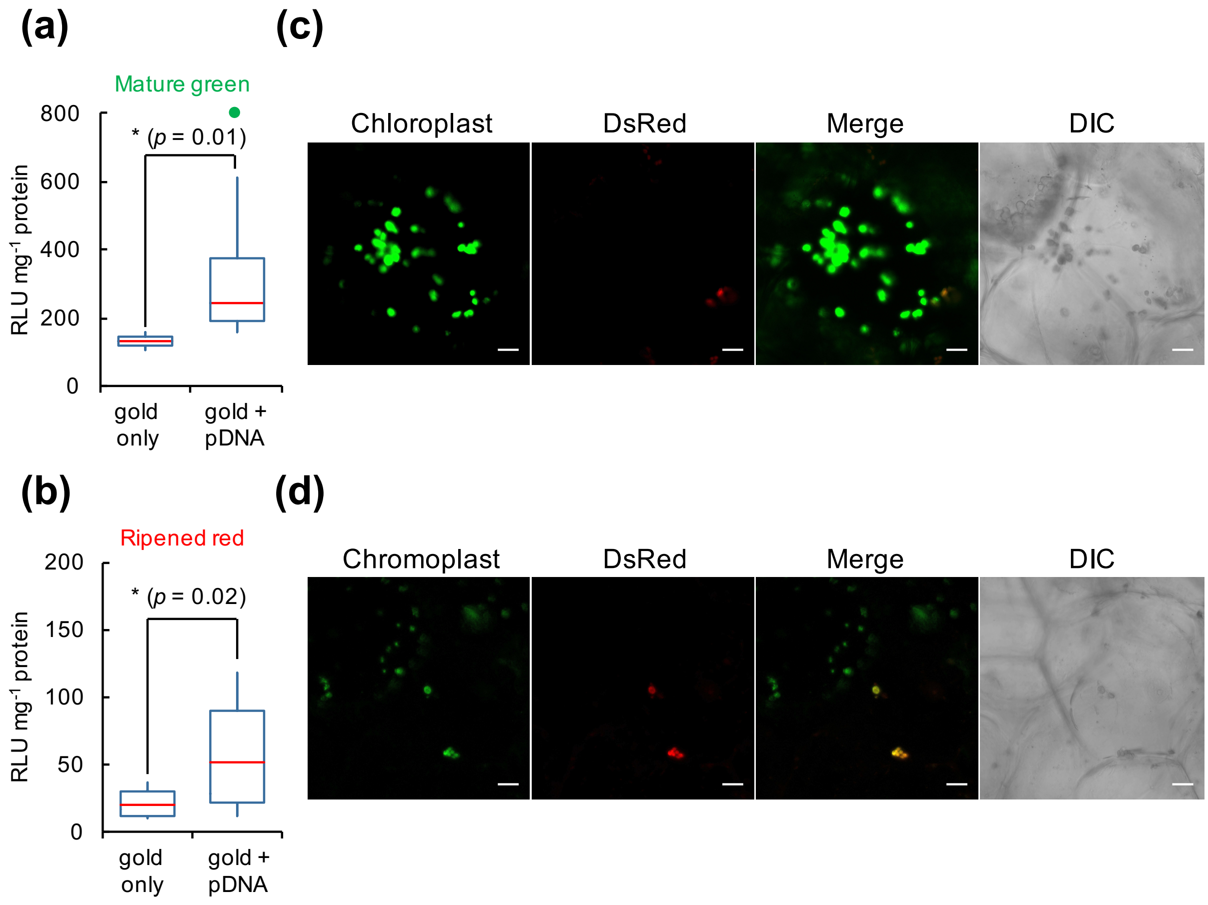


**Figure S19. Expression of reporter proteins in tomato fruit cells mediated by biolistic particle bombardment**.

Luciferase activities in tomato mature green (**a**) and ripened red fruits (**b**) transfected with p*PpsbA::Rluc* by particle bombardment. Gold particles coated with the pDNA or gold particles only were bombarded to tomato fruit slices. Rluc activity in total fruit proteins was assayed at 24 hours post-bombardment. The distributions of Rluc activity in 10 samples ($n$ = 10) were shown as the box plot. Red lines indicate the median of the distribution of data. Green dot represents an outlier in the data distribution. Asterisk represents the significant difference of mean analyzed by Student's *t-test*. (**c**) and (**d**) Expressions of DsRed in plastids in pericarpic cells of mature green (**c**) and ripened red (**d**) tomato fruit slices. Tomato fruit slices were bombarded with p*Prrn*::*DsRed*::*TpsbA*-coated gold nanocarriers and CLSM observation was performed at 24 hours post-transformation. Chlorophyll and carotenoids autofluorescences in chloroplasts and chromoplasts, respectively, were shown by green color. Scale bars = 20 µm.

**Table S1.** Amino acid sequences, molar mass, computed isocratic point (pI) and net charge at pH 7.0 of the cationic domain-fused chloroplast-targeting peptide (CTP) KH$_9$-OEP34, the cell-penetrating peptide (CPP) BP100, and its cationic domain fusion derivatives BP100-KH$_9$ and KH$_9$-BP100. Underlined regions represent the chloroplast-targeting domain in the CTP and the cell-penetrating domain in the CPPs.

<table>
<tr><th>Peptide</th><th>Amino acid sequence</th><th>Molar mass (g/mol)</th><th>Isocratic point (pI)</th><th>Net charge at pH 7.0</th><th>Reference</th></tr>
<tr><td>KH$_9$-OEP34</td><td>KHKHKHKHKHKHKHKHKHMFAFQYLLVM</td><td>3650.4</td><td>11.2</td><td>9.9</td><td>[1]</td></tr>
<tr><td>BP100</td><td>KKLFKKILKYL</td><td>1421.8</td><td>10.9</td><td>5.0</td><td>[2]</td></tr>
<tr><td>BP100-KH$_9$</td><td>KKLFKKILKYLKHKHKHKHKHKHKHKHKH</td><td rowspan="2">3809.7</td><td rowspan="2">11.5</td><td rowspan="2">14.9</td><td>[3]</td></tr>
<tr><td>KH$_9$-BP100</td><td>KHKHKHKHKHKHKHKHKHKKLFKKILKYL</td><td>[4]</td></tr>
</table>

**Table S2**. Plasmid DNA molecules used in this study

| Name | Gene expression cassette | Promoter | Terminator | Vector backbone | Size (kilobase) |
|---|---|---|---|---|---|
| p*PpsbA::Rluc* | *PpsbA::Rluc* | *psbA* promoter | Terminator-less | pMDC107 | 11 |
| p*Prrn::GFP(S65T)::TpsbA* | *Prrn::GFP(S65T)::TpsbA* | *rrn16S* promoter | *psbA-3'UTR* | pUC19 | 3.7 |
| p*Prrn::DsRed::TpsbA* | *Prrn::DsRed::TpsbA* | *rrn16S* promoter | *psbA-3'UTR* | pUC19 | 3.4 |

**Table S3**. Polydispersity index (PDI) of the p*PpsbA::Rluc*/$KH_9$-OEP34 complexes formed at different N/P ratios as measured by DLS. The data are presented as the mean ± SD of three replicates.

| N/P ratio | PDI |
|---|---|
| 0 | 0.957 ± 0.074 |
| 0.1 | 0.697 ± 0.126 |
| 0.5 | 0.341 ± 0.041 |
| 1.0 | 0.552 ± 0.022 |
| 2.5 | 0.471 ± 0.006 |
| 5.0 | 0.525 ± 0.059 |
| 10 | 0.792 ± 0.038 |
| 25 | 0.577 ± 0.021 |

**Table S4**. Polydispersity index (PDI) of the p*PpsbA::Rluc*/$KH_9$-OEP34/BP100 complexes formed at different N/P ratios as measured by DLS. The data are presented as the mean ± SD of three replicates.

| N/P ratio | PDI |
|---|---|
| 0 | 0.275 ± 0.040 |
| 0.1 | 0.342 ± 0.033 |
| 0.25 | 0.349 ± 0.022 |
| 0.5 | 0.218 ± 0.006 |
| 1.0 | 0.405 ± 0.020 |
| 2.5 | 0.443 ± 0.012 |
| 5.0 | 0.401 ± 0.006 |
| 10 | 0.285 ± 0.017 |

**Table S5**. Polydispersity index (PDI) of p*Prrn::GFP(S65T)::TpsbA*/$KH_9$-OEP34 complexes formed at different N/P ratios as measured by DLS. The data are presented as the mean ± SD of three replicates.

| N/P ratio | PDI |
|---|---|
| 0 | 0.558 ± 0.499 |
| 0.1 | 0.894 ± 0.184 |
| 0.5 | 0.447 ± 0.117 |
| 1.0 | 0.386 ± 0.042 |
| 5.0 | 0.383 ± 0.165 |
| 10 | 0.395 ± 0.023 |

**Table S6**. Polydispersity index (PDI) of p*Prrn::GFP(S65T)::TpsbA*/$KH_9$-OEP34/BP100 complexes formed at different N/P ratios as measured by DLS. The data are presented as the mean ± SD of three replicates.

| N/P ratio | PDI |
|---|---|
| 0 | 0.363 ± 0.029 |
| 0.1 | 0.336 ± 0.022 |
| 0.25 | 0.587 ± 0.105 |
| 0.5 | 0.420 ± 0.005 |
| 1.0 | 0.415 ± 0.020 |
| 2.5 | 0.403 ±0.007 |
| 5.0 | 0.390 ± 0.013 |
| 10 | 0.390 ± 0.014 |

**Table S7**. Polydispersity index (PDI) of p*Prrn::DsRed::TpsbA*/$KH_9$-OEP34 complexes formed at different N/P ratios as measured by DLS. The data are presented as the mean ± SD of three replicates.

| N/P ratio | PDI |
|---|---|
| 0 | 0.405 ± 0.454 |
| 0.1 | 0.902 ± 0.169 |
| 0.5 | 0.512 ± 0.046 |
| 1.0 | 0.330 ± 0.163 |
| 5.0 | 0.374 ± 0.014 |
| 10 | 0.631 ± 0.319 |

**Table S8**. Polydispersity index (PDI) of p*Prrn::DsRed::TpsbA*/$KH_9$-OEP34/BP100 complexes formed at different N/P ratios as measured by DLS. The data are presented as the mean ± SD of three replicates.

| N/P ratio | PDI |
|---|---|
| 0 | 0.457 ± 0.082 |
| 0.1 | 0.709 ± 0.031 |
| 0.25 | 0.592 ±0.030 |
| 0.5 | 0.384 ± 0.009 |
| 1.0 | 0.380 ± 0.002 |
| 2.5 | 0.291 ± 0.032 |
| 5.0 | 0.336 ± 0.009 |
| 10 | 0.404 ± 0.014 |

**Table S9**. Primers used in this research. The underlined sequences in the primers represent restriction enzyme digestion sites.

| Primer name | Sequence (5' – 3') | length (bp) | Specific sequence |
|---|---|---|---|
| *PpsbA*_F | TCTAGATCTACATACACCTTGGTTGACAC | 29 | *NtpsbA* promoter |
| *PpsbA*_R | GGATCCGCTTAATTTCTCCTCTTTAGTTCTTGG | 33 | |
| *Prrn*_F | TCTAGAGCTCCCCCGCCGTCGTTC | 24 | *Ntrrn16* promoter |
| *Prrn*_R | ACCATGGGTTCCCTCCCTACAACTCAC | 27 | |
| *Rluc*_F | GGATCCATGACTTCGAAAGTTTATGATC | 28 | *Rluc* gene |
| *Rluc*_R | GAATTCTTATTGTTCATTTTTGAGAACTCGCTC | 33 | |
| *GFP(S65T)*_F | CCATGGATGCGTAAAGGCGAAGAGCTG | 27 | *GFP(S65T)* gene |
| *GFP(S65T)*_R | GGATCCTCATTTGTACAGTTCATCCATACCATGC | 34 | |
| *DsRed*_F | CCATGGATGGACAACACCGAGGACGTC | 27 | *DsRed* gene |
| *DsRed*_R | GGATCCCTACTGGGAGCCGGAGTGG | 25 | |
| *TpsbA*_F | GGATCCTCAAGAGCGATCCTGGCCTAG | 27 | *NtpsbA3'-UTR* (Terminator) |
| *TpsbA*_R | GAATTCTAAATGCAAGAAAATAACCTCTCCTTC | 33 | |

*Nt* – *Nicotiana tabacum*, *3'-UTR* – 3'-untranslated region

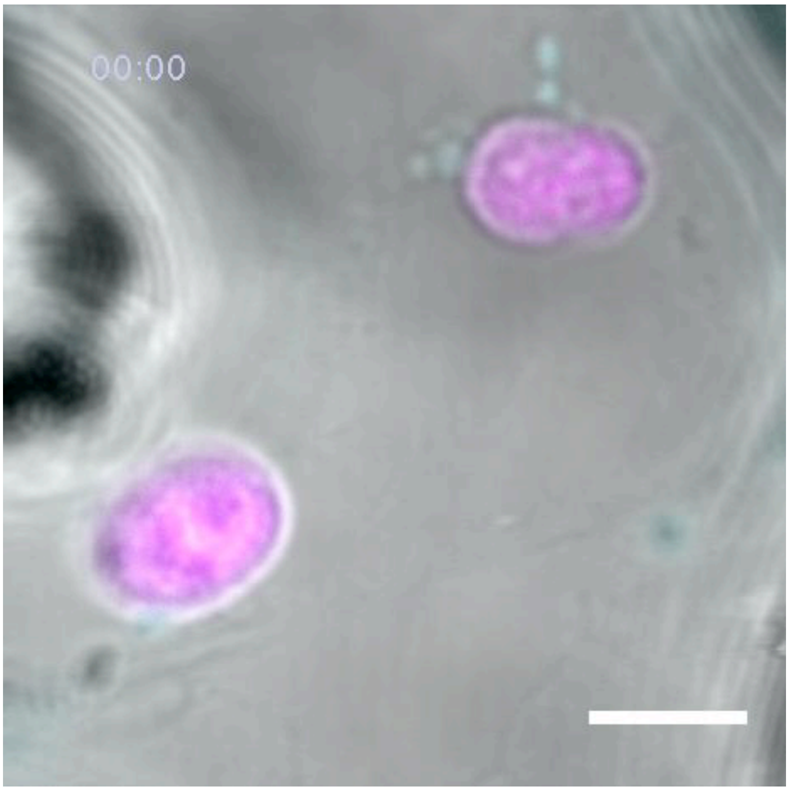


**Movie S1. Intracellular trafficking of pDNA/peptide complexes to chloroplasts.**

Time-course observation of Cy3-labeled pDNA/peptide complexes was performed 2 hours after infiltration of the complexes into *N. benthamiana* leaves. Twenty images were collected within 76 seconds of observation by CLSM imaging. Cyan represents the fluorescent signals from Cy3-labeled pDNA molecules. Magenta represents the autofluorescence signal from chlorophyll in the chloroplasts. Scale bar = 5 µm.

References:

[1] T. Yoshizumi, K. Oikawa, J. A. Chuah, Y. Kodama, K. Numata, *Biomacromolecules* **2018**, 19, 1582.

[2] J. A. Chuah, T. Yoshizumi, Y. Kodama, K. Numata, *Sci Rep* **2015**, 5, 7751.

[3] K. K. Ng, Y. Motoda, S. Watanabe, A. Sofiman Othman, T. Kigawa, Y. Kodama, K. Numata, *PLoS One* **2016**, 11, e0154081.

[4] a) K. Numata, M. Ohtani, T. Yoshizumi, T. Demura, Y. Kodama, *Plant Biotechnol J* **2014**, 12, 1027; b) M. Lakshmanan, Y. Kodama, T. Yoshizumi, K. Sudesh, K. Numata, *Biomacromolecules* **2013**, 14, 10.
